# Persistent microbiome members in the common bean rhizosphere: an integrated analysis of space, time, and plant genotype

**DOI:** 10.1038/s41396-021-00955-5

**Published:** 2021-03-26

**Authors:** Nejc Stopnisek, Ashley Shade

**Affiliations:** 1 Department of Microbiology and Molecular Genetics, Michigan State University, East Lansing, MI 48840 USA; 2 The Plant Resilience Institute, Michigan State University, East Lansing, MI 48840 USA; 3 The DOE Great Lakes Bioenergy Research Center, Michigan State University, East Lansing, MI 48840 USA; 4 Program in Ecology, Evolutionary Biology and Behavior, Michigan State University, East Lansing, MI 48840 USA; 5 Department of Plant, Soil and Microbial Sciences, Michigan State University, East Lansing, MI 48840 USA

**Keywords:** Microbial ecology, Microbiome

## Abstract

The full potential of managing microbial communities to support plant health is yet-unrealized, in part because it remains difficult to ascertain which members are most important for the plant. However, microbes that consistently associate with a plant species across varied field conditions and over plant development likely engage with the host or host environment. Here, we applied abundance-occupancy concepts from macroecology to quantify the core membership of bacterial/archaeal and fungal communities in the rhizosphere of the common bean (*Phaseolus vulgaris*). Our study investigated the microbiome membership that persisted over multiple dimensions important for plant agriculture, including major U.S. growing regions (Michigan, Nebraska, Colorado, and Washington), plant development, annual plantings, and divergent genotypes, and also included re-analysis of public data from beans grown in Colombia. We found 48 core bacterial taxa that were consistently detected in all samples, inclusive of all datasets and dimensions. This suggests reliable enrichment of these taxa to the plant environment and time-independence of their association with the plant. More generally, the breadth of ecologically important dimensions included in this work (space, time, host genotype, and management) provides an example of how to systematically identify the most stably-associated microbiome members, and can be applied to other hosts or systems.

## Introduction

Agriculture requires more efficient use of available resources, and the naturally occurring, soil-dwelling microbiota offers potential to contribute to the responsible intensification of agriculture. Selection and breeding of plants for their beneficial associations with microbiota has promise to deliver a new generation of microbe-improved plants [1–6]. The ideal outcome of such efforts would achieve a balance of sustainable agriculture with food security. To achieve this, we must understand the relationships between plants and their associated microbiomes, including the differentiation of key or “core” members that engage with the plant directly from the members that associate transiently [7].

Common bean (*Phaseolus vulgaris* L.) is the most important food legume grown worldwide, and especially for developing economies in South America, Africa, and Asia [8]. The origin of common bean is central Mexico, and from there it spread to Central and to South America around 165,000 years ago [9]. This resulted in the development of two major and eco‐geographically distinct common bean gene pools with partial reproductive isolation [10–12]. The Mesoamerican gene pool was distributed from northern Mexico to Colombia and the Andean gene pool ranged from southern Peru to northwestern Argentina. Since 8000 years ago, each pool was separately and selectively bred, leading to further diversification between them [9, 13, 14]. Because of pre-existing genetic differences in each gene pool followed by divergent breeding history, common bean presently offers a distinctive opportunity for understanding how the host and the environment contribute to rhizosphere microbiome assembly.

Persistence is the ability of a population to maintain itself within a locality over time (e.g., with a non-zero local abundance). Understanding ecological persistence is important in multiple contexts, including epidemiology and disease eradication, species conservation given climate change, and temporal partitioning of space or resources (e.g., [15–18]). Population persistence can be supported by both ecological and evolutionary mechanisms, including metapopulation dispersal among interconnected spatial patches, diversification or genetic evolution in response to changing conditions, or phenotypic plasticity [16]. Many of these mechanisms have some stochastic contributions. For example, passive dispersal across patches and demographic changes, like births and deaths, can stochastically contribute to population persistence [16]. The rhizosphere microbiome assembles to a host plant from proximal soil, and it is known that some microbiome members are recruited by the host (e.g., nitrogen-fixing rhizobia to nodulating legumes) while other members assemble opportunistically [2]. If considered across a breadth of possible environmental dimensions relevant for host success, the ecological trait of persistence could inform as to which microbiome members engage consistently with a particular plant species.

The objective of this study was to apply approaches and concepts from macroecology to identify the persistent members of a core bacterial, archaeal, and fungal root microbiome inclusive of multiple gradients and categories of drivers expected to be important for plant agriculture. With the cooperation of the U.S. Bean Coordinated Agricultural Project (Bean CAP), we executed a first-of-its-kind study of two divergent bean genotypes grown under field conditions in five major North American bean growing regions, including Michigan, Nebraska, Colorado, and Washington. These two genotypes belong to the Mesoamerican (Eclipse genotype) and Andean (California Early Light Red Kidney, CELRK genotype) gene pools that represent the major divergences from the wild bean ancestor [9]. For these two divergent genotypes, we also assessed how core members of the root microbiome changed over plant development and root compartment. We also used public data to perform a comparative analysis of the bacterial microbiome found in our study with microbiome members detected in other bean genotypes grown in South America [19, 20]. From our effort that was inclusive of both broad biogeography (including the U.S. and Colombia, and with different U.S. management strategies), plant development, and inter-annual plantings, we discovered a stable bean rhizosphere microbiome of 48 members that persistently associated with this nutritionally, agronomically, and economically important crop. This stable “core” was discovered in spite of apparent microbiome differences that were attributable to local soil conditions and management. However, we did not detect an influence of plant genotype, suggesting that this core membership supersedes it.

## Material and methods

### Study design, sampling and soil physicochemical analysis

We designed a biogeography study of two divergent bean genotypes (from Mesoamerican and Andean gene pools [21, 22]), both grown in the field in the summer of 2017 at five research and extension farms that represent major U.S. bean production regions (Table 1) [23]. The research and extension farms were: Saginaw Valley (SVERC), Michigan (MI), Montcalm county (MRF), MI, Scott Bluff county, Nebraska (NE), Fort Collins, Colorado (CO), and Othello, Washington (WA).

**Table 1:** Description of geographical, management and soil properties of the common bean growing locations included in the study.

Sample same	Growing location	Elevation	Bean genotype	Weather (T, precipitation)^a^	Rotation history	Fertilization (per acre)	Irrigation	pH	Nitrogen (%)	Organic matter (%)
SVREC	Saginaw Valley Research and Extension Center, MI, US	190 m	CELRK	24.5 °C, 5.7 mm	Common bean, wheat, maize	Synthetic (400lbs of 15N-5 P-13K)	None	7.7 (± 0.1)	0.13 (± 0.00)	2.33 (± 0.06)
MRC	Montcalm Research Center, MI, US	280 m	Eclipse	24 °C, 7.4 mm	Common bean, maize, potato	Synthetic (200lbs of 19N-0P-19K)	Yes	5.9 (± 0.4)	0.10 (± 0.01)	2.13 (± 0.06)
NE	Scottsbluff, NE, US	1200 m	CELRK and Eclipse	27 °C, 2.18 mm	Common bean, maize, common bean	None	Yes	7.9 (± 0.1)	0.07 (± 0.01)	1.39 (± 0.18)
CO	ARDEC, Fort Collins, CO, US	1536 m	CELRK and Eclipse	31 °C, 1.4 mm^b^	Common bean, maize, barley	Organic (220lbs urea (2016), 3 tons manure (2015))	Yes	8.1 (± 0.2)	0.11 (± 0.00)	1.7 (± 0.15)
WA	WSU, Othello, WA, US	320 m	CELRK and Eclipse	26.3 °C, 0.2 mm	Common bean, maize, wheat	Synthetic (40lbs N/20lbs P/10lbs ZN2O5)	Yes	6.1 (± 0.4)	0.08 (± 0.01)	1.78 (± 0.10)

^a^Temperature (as max air temperature) and precipitation are averaged for the period between May 1st and July 31st 2017.

^b^Data available only for the period between Jun 28th and Jul 31st 2017.

We selected two common bean cultivars with very distinct genotypes: Eclipse [21] of Mesoamerican origin and California Early Light Red Kidney (CELRK), an old kidney bean landrace of Andean origin [22]. Even though we included only two bean genotypes, we selected highly divergent genotypes from far ends of the spectrum of the common bean genetic diversity; these lineages diversified after a biogeographic bottleneck that separated them between Central and South America [24]. We hypothesized that if there was an effect of bean genotype on the root microbiome, it should be measurable when comparing these genotypes from divergent lineages. Both bean genotypes were grown in each growing location, except in Michigan, where CELRK was grown at Saginaw Valley and Eclipse at Montcalm county. Triplicate bean plants of each genotype were grown in each of three (MI, WA, CO), or four (NE) plots, totaling 9 (for MI, WA, CO) or 12 (NE) plants per growing location. Plants were harvested at flowering stage (mid to late July) because we wanted to analyze mature microbial communities and control for potential differences in the microbiome over early plant development.

Plant roots with attached soil were packed into bags, stored on ice and shipped immediately to Michigan State University for processing. From each sample, root-attached soil was collected by gently shaking the roots and designated as rhizosphere soil; unattached root-zone soil was also collected and used as bulk soil. It was expected that the rhizosphere soil contains a subset of microbiome diversity that is recruited from the bulk soil [25] but we note that these soils were not “bulk” in the traditional definition, in that they were collected from the agricultural fields and expected to be proximal to and influenced indirectly by the roots of previous crops. Soils were sieved through 4 mm sieves to remove any plant debris and larger soil minerals before soil physical-chemical analysis. Soil analysis was done at the Michigan State Soil Plant and Nutrient Laboratory on root-zone soil samples pooled by plant genotype and plot (see Supporting Text for details).

We designed a second temporal study to assess the dynamics of the core taxa over plant development. The two bean genotypes, CELRK and Eclipse, were grown at the same two sites in Michigan, U.S., Montcalm county and Saginaw Valley, in the summer of 2018. Plants root systems were harvested at 5 growth stages, including stage 1: V2 (appearance of second trifoliate; *n* = 62), stage 2: V5 (appearance of fifth trifoliate; *n* = 68), stage 3: flowering (*n* = 47), stage 4: pod filling; (*n* = 20), stage 5: senescence (*n* = 12) and stage 6: drying(*n* = 43). At each sampling time, roots were collected and transported on ice to the laboratory for immediate processing. Rhizosphere soil was collected by gently shaking roots, as described above. Any remaining, tightly-attached soil was designated as rhizoplane soil, and was collected by first vortexing roots in 1x phosphate buffer solution (PBS) for 4 min and finally removing the supernatant after centrifugation for 10 min at 8000 *g* and 4 °C. For the temporal study, samples were kept separate by plant and not pooled for downstream analysis. Collected soil was immediately frozen in liquid nitrogen and stored at −80 °C for standard nucleic acid isolations using manual and kit protocols (Fig. S1, Fig. S2).

### Microbiome sequencing and analysis

There were 31 rhizosphere (attached to the root and pooled by plot) and 8 root-zone/bulk (one pooled sample per plot from a particular growing location and plant genotype) soil samples sequenced for microbiome analysis from the biogeography study, and 125 rhizosphere and 127 rhizoplane soil (individual plants) samples sequenced from the plant development study. DNA extractions, including negative controls, were performed as per standard soil protocols (see Supporting Text for details). 16S rRNA gene amplicon and ITS amplicon sequencing was performed at the Michigan State Genomics Core Research Support Facility. We used primers 515f + 806r [26] for bacteria and archaea and ITS1f + ITS2 [27] for fungal communities. One sample (SVERC1 from the biogeography study) was removed due to low quality sequencing output. We processed the 16S rRNA gene amplicons using an open-reference clustering first against the SILVA v128 database [28, 29]. First, raw reads were merged, quality filtered, dereplicated, and clustered into 97% identity operational taxonomic units (OTUs) using the UPARSE pipeline (version 11, [30]). Reads not matching to the SILVA database were used for de-novo clustering at 97% sequence identity. Reference picked and de-novo reads were combined before taxonomy was assigned [28]. Taxonomic annotations for 16S rRNA gene OTU representative sequences were assigned in the QIIME 1.19 environment [31] using SILVA database [29]. ITS OTU representative sequences were taxonomically annotated using the CONSTAX tool [32] with the UNITE database version 7.2 [33]. OTUs not classified as fungi, OTUs with unassigned taxonomy at the domain level, and OTUs annotated as mitochondria or chloroplasts were removed. Contaminant OTUs were removed using decontam package in R [34]. Additionally, we performed zero-radios OTU analysis (aka ZOTUs that are clustered at 100% sequence identity) for reads detected within the core OTUs. In short, first we subset all reads that were included within the core OTUs and processed them through the UNOISE3 pipeline [35] to create ZOTUs. Then we determined each ZOTU’s affiliation to their originating core OTU by matching the read identities between ZOTU and OTU clusters using a customized code (see GitHub repository).

Statistical analysis, including abundance-occupancy analysis to detect a core microbiome [7], Sloan neutral model fit [36, 37], and data visualization were performed in R (see Supporting Text). The quantification of assembly processes was done using iCAMP [38]. The co-occurrence network and global network properties were calculated using the Molecular Ecological Network Analysis Pipeline (MENAP) [39] and visualized with Cytoscape v.3.5.1 [40]. Comparative analyses with published datasets from Pérez-Jaramillo et al. [19, 20] (BioProject ID PRJEB19467 and PRJEB26084) were performed by downloading study raw reads from NCBI and processing as per our data pipeline to analyze both sets identically. An expanded description of all ecological statistics, MENAP parameters, and our combined core microbiome analysis with public data are provided in Supporting Text.

### Data and code availability

The raw sequence data are deposited in the NCBI Sequence Read Archive (BioProject ID PRJNA524532 (2017 biogeography study; including 16S rRNA and ITS amplicons) and PRJNA606063 (2018 development study). All read processing steps, bioinformatic workflows, R code, and custom scripts are available on GitHub (https://github.com/ShadeLab/PAPER_Stopnisek_2019_BeanBiogeography).

## Results and Discussion

### Sequencing summary and microbial diversity across growing regions

There were 31,255 to 506,166 and 22,716 to 252,810 reads per sample for 16S rRNA and ITS biogeography datasets, respectively. We rarefied samples to 31,255 reads for 16S rRNA gene amplicons and to 22,716 for ITS. With these thresholds, we achieved richness asymptotes for both datasets, suggesting that sequencing efforts were sufficient to capture comparative dynamics and diversity (Fig. S3). The total richness observed at this rarefaction depth was 1,505 fungal and 23,872 bacterial and archaeal OTUs.

As reported in other rhizosphere studies, the total fungal diversity was lower than bacterial/archaeal diversity in the rhizosphere of the common bean [41–43]. Richness varied by growing location (ANOVA, F value = 12.4, *p*-value < 0.0001 and F value = 13.1, *p*-value < 0.0001 for 16S rRNA and ITS data, respectively, Fig. S4) but was highest at the Montcalm Research Farm (Michigan, US) for both, bacteria/archaea and fungi. An analysis of community beta diversity revealed strong biogeographic patterns in community structure explained by location, soil pH and fertilization, in agreement with other literature [44–47] (Fig. S5, Table S1). However, an influence of plant genotype was either weak or not detected, which we partially attribute to the larger number of variables that influence plant growth in field conditions (as opposed to controlled environmental chamber or greenhouse conditions). Our finding generally agrees with observations made in other plant species grown under field conditions, in which plant genotype is reported to have minimal value in explaining microbiome variation. [44, 45, 48] (Fig. S4, Table S2).

The common bean rhizosphere microbiome included the major expected lineages for both bacteria and fungi (Fig. S6), in agreement with other plant rhizosphere studies [49–54]. Together with previous studies, these data provide more evidence that root-associated microbial taxa are phylogenetically and potentially functionally conserved [55]. Proteobacteria, Acidobacteria, Bacteroidetes, and Actinobacteria collectively comprised on average 73.5% of the bacteria/archaeal community, Ascomycota dominated the fungal community with a mean total relative abundance of 53% with notable sample-to-sample variance (range from 16.5% to 84.5%).

### A persistent “core” rhizosphere microbiome is detected across U.S. bean growing regions

**Fig. 1:**
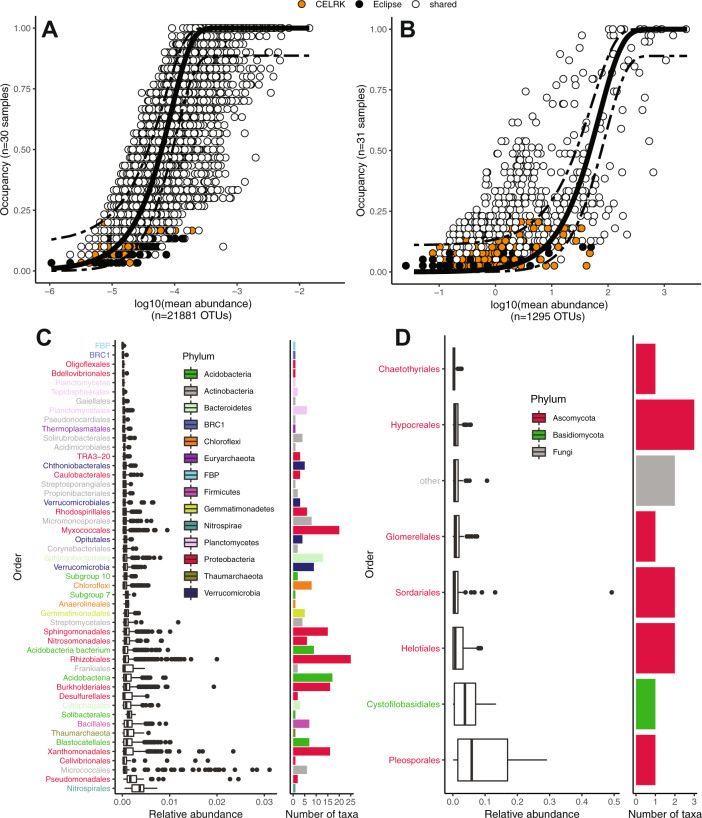
A high number of microbes are consistently detected in the common bean rhizoshere across U.S growing locations. Abundance-occupancy distributions were used to identify core members of the rhizosphere microbiome for bacteria/archaea (**A**) and fungi (**B**). Taxa exclusive to a genotype are indicated in orange (CELRK) or black (Eclipse), and taxa shared across both genotypes are white. The solid line represents the fit of the neutral model, and the dashed line is 95% confidence around the model prediction. Taxa with an occupancy of 1 (i.e., detected in all samples) were considered members of the core. Relative abundance of these taxa is represented as boxplots, grouped by order and number of taxa therein (**C**, **D**). Panels C and D are color-coded by phylum.

We noticed a large number of OTUs that were shared among all growing locations for the bacterial/archaeal communities (2,173 taxa, mean 31.5%, range 29.5% to 34.7%). There was a smaller but notable overlap for the fungal communities (70 taxa or mean 4.5%, range from 0.9% to 17.9%; Fig. S6). These data suggested that, despite measured edaphic differences across growing locations and strong biogeographic signal, the common bean rhizosphere recruited many similar taxa that could be functionally important for the bean. To identify microbial taxa that show the most robust and persistent associations with the plant host, we explored abundance-occupancy distributions of taxa ([56, 57] and references therein) to infer the “core” bean microbiome, which, in our stringent approach, included all taxa with an occupancy of 1 (i.e., found in all soil samples, all plots and across all growing locations; Fig. 1A, B). Among bacteria and archaea, 258 taxonomically diverse taxa were cosmopolitan in the dataset (Fig. 1C), including numerous and abundant Proteobacteria (117 OTUs) with a dominant taxon classified as *Arthrobacter* sp. (FM209319.1.1474, mean relative abundance of 1.43%). The bacterial/archaeal core also contained taxa of interest for potential plant benefits (e.g., *Sphingomonas*, *Rhizobium*, *Bacillus, Streptomyces*), as well as some genera that can be associated with disease (e.g., *Ralstonia*). There were 13 taxa in the fungal core (Fig. 1D), and these were largely composed of Ascomycota (10 OTUs), with dominating taxon OTU823 from the *Phaeosphaeriaceae* family (mean relative abundance 10.1%). Notably, taxa that were unique to either bean genotype were relatively rare and inconsistently detected (Fig. 1, orange and black points). Together, these results suggest that common bean consistently recruits particular microbiome taxa.

Next we wanted to investigate if the prioritized core taxa are indeed selected by the plant environment or assembled through neutral processes by applying the Sloan neutral model [36, 58]. The neutral expectation of abundance-occupancy distributions is that very abundant taxa will have high occupancy, while rare taxa will have low [36, 57–59]. Taxa that deviate from the neutral expectation are more strongly influenced by deterministic factors, like environmental conditions, than by stochastic factors, like drift and dispersal. The neutral model fit of the abundance-occupancy distribution (solid line, Fig. 1A, B) identified several taxa that had frequencies either above or below the 95% confidence intervals of the model (dashed lines). Specifically, 13.7% of the bacterial/archaeal and 30.4% of fungal taxa, deviated from the neutral expectation (Table S3). One hundred and seventy-one out of 258 core taxa were predicted above the neutral model partition; these deterministically-selected taxa are prime candidates for follow-up studies of interactions with the host plant. Notably, this group of taxa that fall above the neutral prediction will include those that engage directly with the host and also those that are fit in the plant environment but do not necessarily have direct engagement; additional experimental work is needed to understand the precise deterministic factors at play. Overall, the bacteria/archaea community had better fit to the neutral expectation than fungal (R^2^ of 0.74 and 0.34, and migration rates (m) of 0.301 and 0.003, respectively), suggesting that dispersal was relatively less limiting for the bacteria/archaea than for the fungi. This finding agrees with other work suggesting that fungi are more sensitive to local climate or more dispersal limited than bacteria [60–63].

Notably, the neutral model method that we have applied here has been criticized as having the potential to overestimate the statistical importance of stochastic processes in shaping community outcomes [38, 64, 65]. Therefore, it is possible that some of the identified taxa that fall within the neutral prediction may be deterministically selected by the plant environment. To investigate this, we used the iCAMP tool to infer community assembly mechanisms by phylogenetic bin-based null model analysis [38]. Results show that deterministic processes may be relatively more important than suggested with the neutral model, ranging between 32.4% and 47.6% (Fig. S7). The deterministic processes for core taxa were only attributable to homogenizing selection, and not heterogeneous selection; this outcome would be consistent with host filtering or recruitment. In addition, there are other methods, such as source-sink modeling [56] and other model expansions that could be further applied, with more datasets, to enrich insights as to the mechanisms of community and population-level drivers (e.g., [66]). More work is needed to determine this, but the take-home point is that both deterministic and neutrally-predicted “core” taxa that have stable associations with the plant should be followed up to test the hypothesis that they are key functional players in the bean microbiome.

### A core rhizosphere microbiome is detected for common bean grown on different continents

**Fig. 2:**
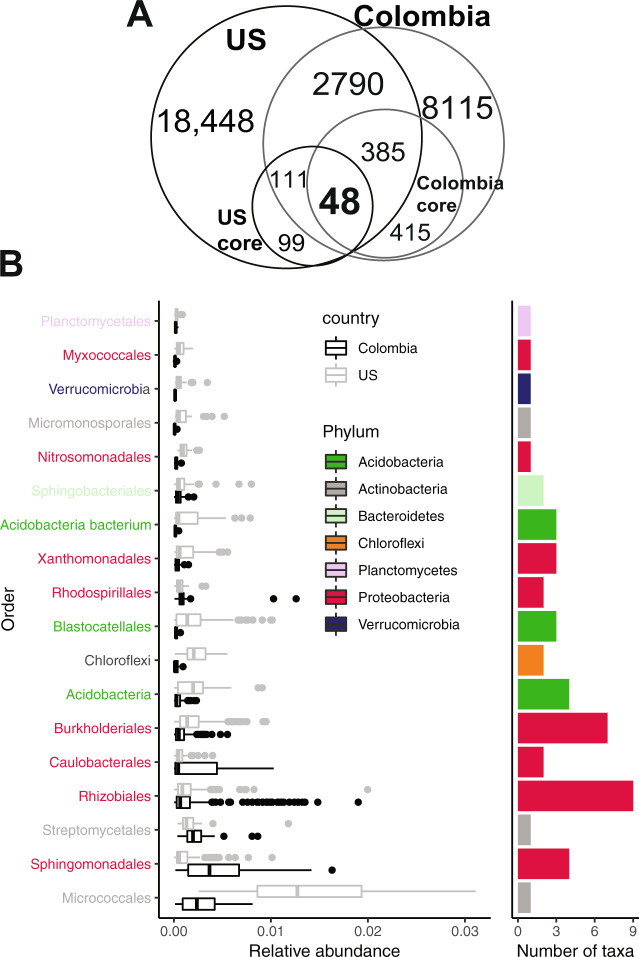
A global core rhizosphere microbiome. There were 3361 shared bacterial/archaeal taxa across U.S. and Colombia rhizosphere samples, suggesting highly similar recruitment across continental scales. Forty-eight taxa were detected in all samples of both datasets, as depicted by the Venn diagram (**A**), and included many Proteobacteria (**B**). Relative abundance of the 48 U.S.-Colombia core taxa is represented as boxplots (left panel), grouped by order and dataset (Colombia/U.S.). Number of taxa per order is represented as bars (right panel). Labels on the y-axis and bars are color-coded by phylum level.

We wanted to better understand if these U.S. core taxa were associated with the bean rhizosphere across a larger geographical scale, which would suggest the potential for selective plant recruitment and cosmopolitan distribution of core taxa. Therefore, we compared our U.S. data to a published study of rhizosphere bacteria and archaea from common beans grown in Colombian agricultural soil [19]. More details of the published study are provided in the supplemental materials. The Colombian study offered a key contrast because it included eight divergent bean lineages, including wild (*n* = 2), landrace (*n* = 1), and cultivated genotypes (*n* = 5), grown in soil from a different continent that has starkly different climate and management from the U.S. growing regions. To enable direct comparison, we re-analyzed raw reads and compared the datasets by matching to either the same taxon identifiers when clustered to SILVA database, or 100% identity by BLAST to de novo clustered reads (see Supplementary Text). Surprisingly, 39.6% (3,359 OTUs) of rhizosphere taxa from the Colombian-grown beans were also shared with the U.S. dataset (Fig. 2). Both datasets included taxa that were highly represented in the other: 62% of U.S. core (159 out of 258) were found also in Colombia, and 51% of Colombian core (433 out of 848) were shared with the U.S. (Fig. 2A). Core taxa were again defined stringently with an occupancy of 1, and 48 taxa were found across all samples, inclusive of both datasets. We refer to this as the “global” core to distinguish the subset from the larger group of core taxa inclusive to the US only (though note that this descriptor is for simplicity and that this does not include global representation of bean root samples on Earth). These global core taxa were composed of many Proteobacteria, with *Rhizobiales* showing the most consistent relative abundance between the studies (Fig. 2B, e.g., 0.187% and 0.138% in Colombia and U.S. dataset, respectively). None of these global core taxa were universally detected in very high abundance, and all but two OTUs (a U.S. *Arthrobacter* sp. and a Colombian *Austrofundulus limnaeus* with mean relative abundances of 1.43% and 1.01%, respectively) would be classified as rare by a typical mean relative abundance threshold below 1%, hinting to a possible role of some rare taxa in providing key functions. A similar observation was made with rhizosphere microbiota of 19 herbaceous plant species, in which taxa of low abundance were among those significantly enriched in the rhizosphere as compared to the bulk soil [67]. Furthermore, results from iCAMP (*n* = 25 OTUs, Fig. S7) and neutral model (*n* = 19 OTUs) predict that homogeneous selective assembly processes influence taxa that belong to the core. Notably, only 48% of these global core taxa have genus classification, suggesting that most are under-described in their functional potential and interactions with plants (Table S4).

We also analyzed reads associated with the global core taxa with the UNOISE3 pipeline [35] to generate predicted biological sequences (zero-radius OTUs–ZOTUs with100% sequence identity) and provide the maximal possible biological resolution. We found that the global core taxa consisted of 422 ZOTUs, and that there was a range of 2 to 35 ZOTUs identified within each OTU (Fig. S8). With one exception (HQ597858.1.1508), all of core OTUs (clustered at 97% sequence identity) contained at least one ZOTU that also had an occupancy of 1. In addition, all of the ZOTUs with an occupancy of 1 were also the most abundant ZOTUs within each OTU (Fig. S8). This result suggests that the same members constitute the core even with increased taxonomic resolution.

A recent study considered the effect of the common bean domestication history on the root microbiome and identified a core set of microbial taxa that are consistently present with these diverse bean genotypes, including bacterial taxa recruited from agricultural and natural soil from Colombia as previously reported [20]. This core also had high representation of *Proteobacteria*, *Acidobacteria*, *Actinobacteria* and *Verrucomicrobia*, similar to those observed in the present study. We also re-analyzed these data and show that 46 (out of 48) of the global core taxa identified here have occupancy > 0.9 across all three included studies (the present study, Pérez-Jaramillo et al [19]. and Pérez-Jaramillo et al [20]). Forty-two of these taxa had the highest possible occupancy of 1, but only within rhizosphere samples from agricultural soils (Table S5). When incorporating data from beans grown in forest soils, the average occupancy decreases to 0.77 (median 0.75, min = 0.53). However, 14 taxa still had an occupancy of 1 when including beans grown in forest soils (Table S5).

In summary, these results show that common bean can associate with a persistent set of rhizosphere microbiome members at taxon and ecotype levels, across diverse bean genotypes, management practices, and across soils from different continents. Additionally, these persistent (“core”) taxa are likely enriched by the host in managed soils, as suggested by their higher occupancy in agroecosystems than in the native soils considered in our meta-analysis and in agreement with the original comparison between agricultural and forest soils by Perez-Jaramillo et al [20].

### Core taxa are enriched in the rhizoplane and are consistently detected across bean development

To identify the common bean core taxa over space (a biogeographic core) we sampled plants across growing locations at the same growth stage (flowering) and focused on the rhizosphere compartment. However, the question remained as to whether these core taxa are detected beyond that particular plant developmental stage. To answer this question, we conducted a field experiment to assess the core taxa over plant development. In the next growing season (2018), we used the same divergent bean genotypes grown at both Michigan, U.S, locations (Montcalm and Saginaw Valley; see Material and Methods). We harvested root systems at 5 different plant development stages including flowering stage. We investigated the relative abundance of the global core taxa and the U.S.-specific core taxa in both rhizosphere (soil that could be removed from the root after shaking, *n* = 125 samples) and rhizoplane (soil adhered to the root tissue and removed via vortex in buffer, *n* = 127 samples) compartments to determine their ability to closely associate with the plant tissue. The range of the rhizosphere sequencing depth was 7,905–78,436 reads per sample, and for the rhizoplane it was 32–189,433 reads per sample. We rarefied to 15,000 reads per samples (i.e., samples reaching richness asymptote), resulting in loss of 3 rhizosphere and 7 rhizoplane samples for a final dataset of 122 rhizosphere and 120 rhizoplane samples. The total richness observed was 36,022 bacterial and archaeal OTUs.

**Fig. 3:**
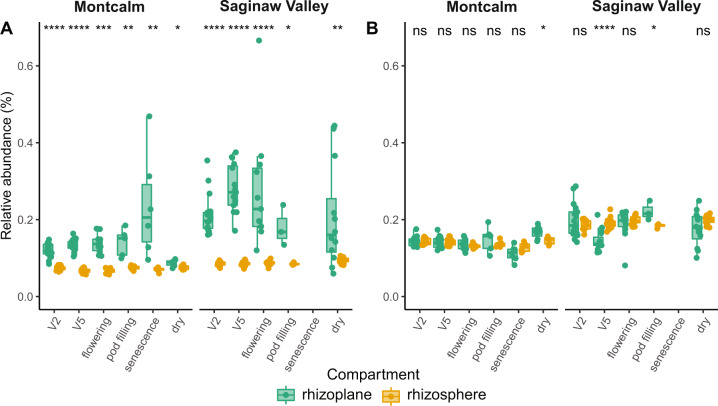
Relative abundance of core taxa in the root system of the common bean during plant development by root compartment and growing location. The combined relative abundance of 48 core taxa is significantly higher in the rhizoplane (green) compared to the rhizosphere (orange) and does not show plant development dependence (**A**). The combined relative abundance of remaining core taxa specific to the U.S. (n = 210), tend to be high throughout the plant development but are equally abundant in both compartments (**B**). Stars above box plots represent statistical significances as determined by Wilcoxon test (**** ≤ 0.0001, *** ≤ 0.001, ** ≤ 0.01, * ≤ 0.05).

From this development time series, we found that all 48 global core taxa were detected a year later on these two Michigan farms. The collective relative abundances of the global core taxa were significantly higher in the rhizoplane as compared to the rhizosphere irrespective of the plant development stage, bean genotype and growing location (Fig. 3A). Interestingly, the remaining US core taxa that were found exclusively in the U.S. dataset at an occupancy of 1 were equally abundant in the rhizosphere and rhizoplane at both Michigan growing locations (Fig. 3B).

**Fig. 4:**
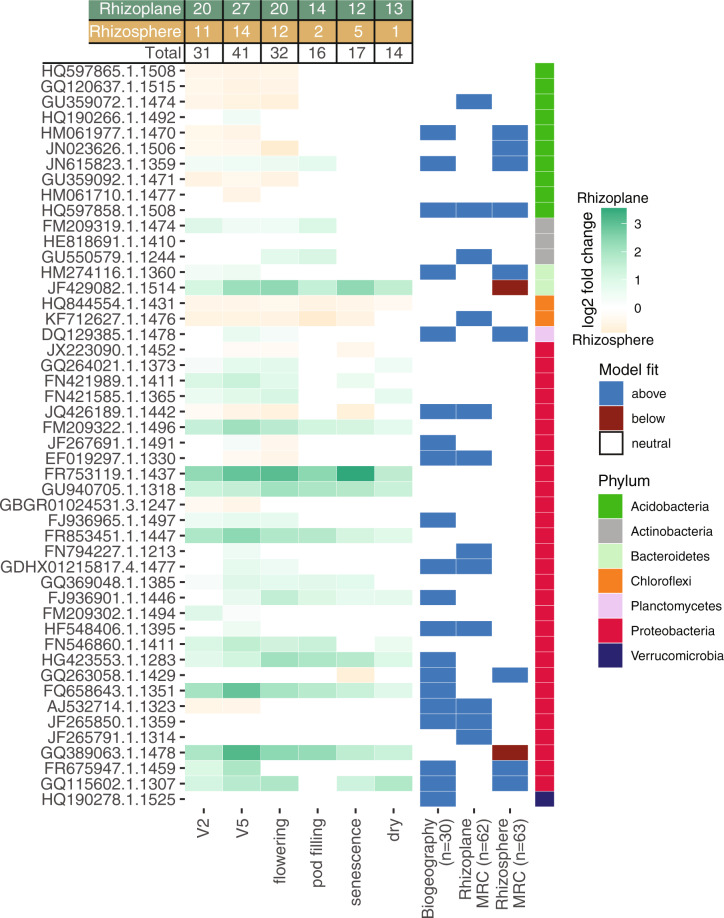
A table summary and heatmap representation of the compartment enrichment of core microbial taxa during plant development, and their agreements with the neutral expectation of abundance and occupancy as determined by the Sloan model. This figure was generated using the 16S rRNA gene amplicon sequencing data from the biogeography dataset, and the 16S rRNA gene amplicon sequencing data from the plant development dataset (Montcalm growing location). DESeq2 [92] was used to identify differentially abundant taxa by compartment (*p*-value < 0.05 after false discovery rate correction). Log2 fold change values were used to determine shading; taxa shaded in green were enriched in the rhizoplane and taxa shaded in brown were enriched in the rhizosphere. Sloan neutral models were fit to both datasets to understand which core taxa were above (blue) or below (red) the model expectation. All taxa that are not highlighted follow the neutral prediction. The sample sizes are provided in brackets. Core taxa are arranged and color-coded by taxonomic classification at the phylum level.

We next asked whether there were enrichments of particular core taxa by plant developmental stage or root compartment (Fig. 4). We used differential abundance analysis and assessed the neutral model prediction of the core taxa at different growth stages and across compartments (Fig. 4). There were minimum 14 and maximum 41 different core OTUs that exhibited preference for either rhizosphere or rhizoplane in a particular growth stage. There were taxonomic signatures in the patterns, as members from the same family had similar compartment preferences. Members of the global core that were Proteobacteria and Actinobacteria were among those taxa enriched in the rhizoplane, while Acidobacteria and Chloroflexi had enrichment in the rhizosphere. Interestingly, enriched taxa to either compartment did not switch preference throughout plant development, with one exception (JF267691.1.1491, an *Acidibacter*). Despite these nuances, all core taxa were consistently found with high occupancy inclusive of the plant development series (Fig. S9). On balance, almost all core taxa showed some growth stage preference, but these trends were specific to each growing location and root compartment. Finally, with two exceptions, deterministically selected core taxa in either rhizoplane or rhizosphere compartment were above the neutral model prediction, suggesting selection by the plant environment (Fig. 4).

Together these results suggest that these core taxa are selected by the plant environment early in development and maintained. Enrichment of the core taxa in the rhizoplane further supports the hypothesis that these core taxa engage closely with the host plant.

### Core taxa are not hub or connector taxa in an inter-domain microbiome network

**Fig. 5:**
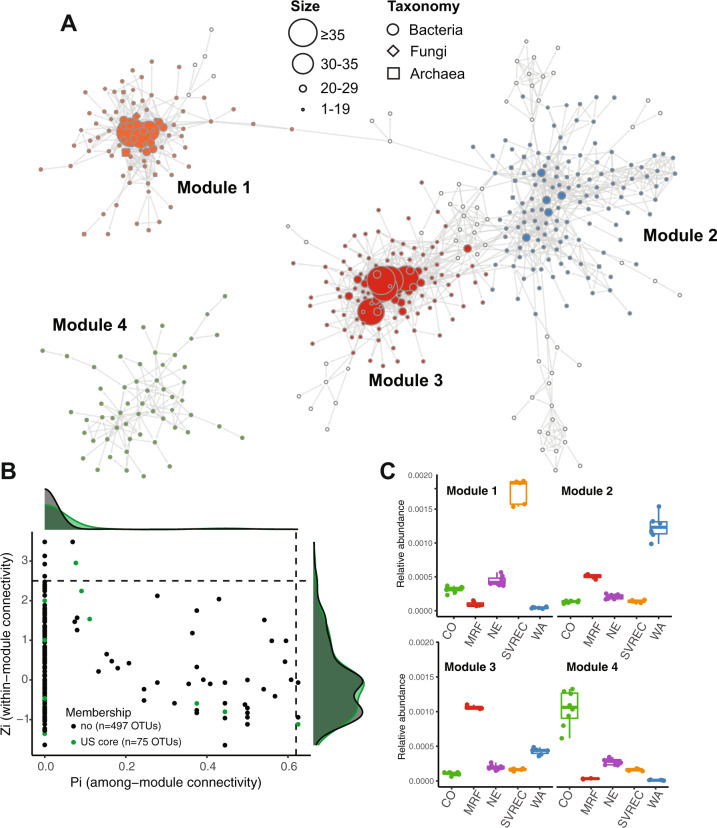
Network co-occurrence analysis shows that core rhizosphere microbiome members are predominantly classified as peripheral taxa that are weakly connected, and generally clustering by the growing location. The network depicts only clusters of modules that were connected by more than 6 nodes (**A**). Nodes shape is representing domain associations (archaeal, bacterial or fungal) and node size is proportional to its total number of connecting edges (**A**). The four largest modules are generally reflective of community biogeography and distinguished by color (A). The within- (Zi) and among- (Pi) module connectivity plot was used to identify module (Pi < 0.62, Zi > 0.2.5) or network hub taxa (Pi > 0.62, Zi > 2.5), as well as connector (Pi > 0.62, Zi < 2.5) and peripheral taxa (B). The density plots surrounding the Zi-Pi plot represent core (green) and non-core taxa (black) (**B**). The relative abundances of taxa within each module is represented in box plots (**C**).

Network analysis has been proposed to be a useful method to identify important members of the plant microbiome with beneficial traits [68–70]. Hub taxa, identified by their high connectivity with many members of the community, are regarded as the most important part of the community and influence network structure and community stability [71, 72]. We applied this method to ask if any of the core taxa that we identified using abundance-occupancy were also key for co-occurrence network structure. Additionally, we were interested in identifying fungal-bacterial co-occurrences because of reports of their potential benefits for the plant [73, 74]. To explore these patterns we applied the molecular ecology network analysis pipeline (MENAP) which constructs ecological association network through random matrix theory (RMT) [39]. We merged rarified 16S and ITS rhizosphere datasets from the biogeography study, filtered the datasets to include taxa with occupancy greater than or equal to 50%, and considered only correlations significant at *p*-value < 0.05 and RMT threshold of 0.88. The resulting network included 572 taxa (nodes) and 1,857 statistically significant co-occurrences (edges) structured among 52 modules (Fig. 5). Most of the modules were relatively small, with only six including more than 10 nodes (Fig. 5A). The network was scale-free and had small-world characteristics as indicated by the node degree distribution fitting to the power law model (R^2^ = 0.993), and also had significant deviation of the modularity, length and clustering coefficients from those calculated from random network (i.e., constructed from same number of nodes and edges), respectively (Table S6).The majority of correlations were bacterial-bacterial (rather than bacterial-fungal or fungal-fungal, Fig. 5B).

The topological role of each taxon within the network was determined by the relationship between their within-module (Zi) and among-module (Pi) connectivity scores as described in [75]. Based on this, the majority of taxa were peripheral (potentially, specialists; 563 nodes) (Fig. 5B). There were also 3 connectors and 6 module hubs, but no network hubs. Indeed, in agreement with the beta-diversity analysis (Fig. S5, Supporting Materials), the four largest modules had strong geographic signal (Fig. 5C). We note that cross-domain edges constituted only a small fraction of all co-occurrences (*n* = 168; bacteria-fungi = 156, archaea-bacteria = 6 and archaea-fungi = 6).

The analysis identified 26 co-occurrences between core taxa. However, we were surprised to find that only two bacterial core and no fungal core taxa were also classified as module hubs (taxa that connect to many other taxa within a modules) or connectors (taxa that connect across modules). As exceptions, core *Chitinophagaceae* taxon FR749720.1.1492 was a module hub node and a *Nitrobacter* sp. GDHX01215817.4.1477 was a connector. Our results, inclusive of a dataset of divergent plant genotypes and broad biogeography, suggest that while hub and connector taxa may be important for the structure and dynamics of the generally root-associated microbial community, these taxa are not consistently detected in the common bean rhizosphere and, by deduction, could not be of universal importance for the host plant. Our study cannot speak to the potential for functional redundancy among hub or connector taxa, which could ultimately suggest a functional core among phylogenetically diverse taxa [76]. Taken together, these results suggest that core taxa likely are important for the plant, while hub and connector taxa are important for the integrity of the soil microbial community and its responses to the local environment. Again, amplicon sequence data cannot speak directly to function and so hypotheses as to their importance for the host plant must be tested for the identified core set of taxa.

Numerous studies have aimed to detect core microbiome members for various plants and animals (see references within [7, 77]), including, recently, across compartments of the common bean [78]. Because methods are inconsistent across studies and because the parameters used to define a core are often arbitrary, there is some question as to how robust or useful such studies may be. For example, in the study of bean compartments [78], the core was defined as taxa detected across all plant compartments, but not necessarily in every sample; this core definition would include multiple spuriously detected taxa and therefore increase the perceived relative importance of stochastic assembly. Given the differences in definitions and objectives across studies, it has become easy to overlook new studies that claim to have discovered a core microbiome for their system.

In our prior work, we discussed methods for ecologically informing core microbiome members from diverse datasets using abundance-occupancy distributions, and for determining thresholds of each dimension based on spatial and temporal study designs. Here, we applied those concepts to prioritize, for the first time, a core plant microbiome that stably persists at across growing locations on two continents, over plant development, over two years of annual plantings at two different farms, across highly divergent plant genotypes, across management practices, and also across datasets collected by different research groups. Furthermore, core members were collectively enriched in the rhizoplane, and not just the rhizosphere. These multiple and consistent lines of evidence provide strong support that these taxa are likely to engage with the plant

We took a multi-step approach to identify persistent taxa and assess their detection over space, genotype, and time. First, we began with collection and analysis of broad biogeographic dataset (inclusive of two divergent host gene pools) to conservatively identify the most persistent taxa associated with bean. The spatial design was balanced across the major growing regions such that each contributed equally to the analysis, preventing bias towards taxa prevalent at some locations but not others. We reasoned that more environmental variability should be captured across broad geographic scales [79–83], rather than over the temporal fluctuations of a single location. Therefore, it was important to assess a balanced dataset that included the greatest range of environmental conditions expected to drive microbial communities. The observation of an occupancy of 1 (100% detection) for dozens of microbial taxa is notable and unexpected given the geographic range, differences in soil properties, and diverse management practices included among the growing locations. Then, with those spatially persistent taxa identified, we next asked if they were relevant within the context of rhizosphere development at Michigan growing locations. It could have been that taxa prioritized as persistent at broad geographic scales were not detected over time. However, not only were these same persistent taxa detected, but they were also collectively enriched in the rhizoplane, which strongly suggests an association with bean. The consistent detection of the prioritized taxa from the biogeography study within the development study demonstrates that our general approach has utility to inform across spatial and temporal dimensions of microbiome dynamics.

A majority of the 48 core bacterial taxa among these common bean rhizospheres are under-described or largely unknown, as only 23 out of 48 have a genus-level classification. But, due to their ubiquitous association with the bean over space and time, we hypothesize that these taxa provide functions that are important for common bean health and should be targeted for additional study and microbiome management in support of common bean productivity and wellness. To test this hypothesis further research is needed, but plant-beneficial traits have been previously reported for members of the genera that we identified as part of the bean core microbiome and even more so for those enriched in the rhizoplane. For example, members belonging to the genera *Mesorhizobium* and *Rhizobium* are known symbiotic nitrogen fixers of legumes [84, 85], and *Ramlibacter* sp. have the ability to promote P mobilization [86]. On the other hand *Arthrobacter* sp. have been shown to be involved in production of phytohormones such as auxins, gibberellins, and cytokinins [87]. Similarly indole acetic acid production was found for species of *Novosphingobium* and *Sphingomonas* [88, 89]. *Caulobacter* sp. has been frequently detected with many plant hosts either in the rhizosphere or endosphere, but it is only recently that their plant growth promoting traits have been described [44, 49, 90].

Discovering these persistent taxa is a first step in a rich line of inquiry to understand host engagement with them. The next steps are to understand any functions associated with these taxa and to determine whether and how they contribute to plant health and productivity under different growth conditions, such as drought or with particular management strategies [48, 91]. These steps will include cultivation dependent and independent approaches aimed to enrich and isolate core members, assemble or bin genomes from isolates and metagenomes, annotate functional genes on both chromosomes and plasmids, link functions and activities through transcript or metabolome analyses, and perform experiments with constructed communities of core members to test hypotheses about microbiome engagement with and benefits to the plant. It could be that some of these taxa do not directly engage with the plant or provide any benefit, but, due to their stable association with the plant, it is likely that some of them do.

To conclude, this work provides approaches and insights for prioritizing stable and persistent microbiome members as “core”, inclusive of multiple ecologically relevant dimensions that drive plant-microbiome relationships. This work advances goals in plant-microbiome management and microbe-improved crops by providing insights into core member persistence, identities, and dynamics.

## Supplementary information

Supplemental Material

Table S4

Table S5

Table S6
