# Supplementary material for: Persistent microbiome members in the common bean rhizosphere: an integrated analysis of space, time, and plant genotype
Source: ISME J. 2021 Mar 26;15(9):2708–22. doi: 10.1038/s41396-021-00955-5 (PMC8397763; doi:10.1038/s41396-021-00955-5)
Supplement: Supplementary file 1 — Supplemental Material [file 41396_2021_955_MOESM1_ESM.pdf]

Supporting Text for

Persistent microbiome members in the common bean rhizosphere: an integrated analysis of space, time, and plant genotype

Nejc Stopnisek and Ashley Shade

Supporting Results and Discussion

Beta diversity: strong biogeographic but weak plant genotype influence on microbiome dynamics

As expected, there were differences across growing locations in edaphic soil properties (e.g. pH, nitrogen species, organic matter) as well as in management practices (e.g. fertilization, irrigation, crop rotation) and climate (**Table 1**). Growing location, pH and fertilization explained differences in microbial community structure for bacteria/archaea and fungi, but plant genotype did not (**Fig. S5**, **Table S1**). Fungal and bacterial/archaeal communities had synchronous biogeographic patterns (Procrustes m12 squared: 0.2137, R^2=0.8867 and *p*-value=0.001), supporting that both communities are shaped by local edaphic factors. An effect of growing location on beta-diversity has been previously reported for plant- and soil- associated microbiomes [1–4].

We selected two distinct bean genotypes with divergent evolutionary and breeding histories to assess the impact of plant genotype on the root microbiota, expecting that any signal would be maximized between these lineages. There was a weak interaction between plant genotype and growing location in explaining fungal community structure (PERMANOVA, R^2=0.0377, *p*-value=0.001) (**Table S1**), but no effect of plant genotype alone on the bacterial and archaeal communities, even when controlling for growing location. Similarly, plant genotype had a weak but statistically detectable effect on bacterial and archaeal richness (*p*-value=0.019) but no measurable effect on fungal richness (*p*-value=0.95) (**Fig. S4**). An analysis of differential

taxon abundances across genotypes (DESeq2 [5]) detected 20 taxa out of 21,881 total that were distinguishing between the genotypes CELRK and Eclipse, but their adjusted *p*-value were marginally significant (p-value> 0.02; **Table S2**). The absence of a robust genotype effect on the microbiome is in contrast to a previous study on common bean [6]. However, several studies have reported a relatively weak to no influence of genotype more generally on the rhizosphere communities of other plants grown in the field [1, 2, 7]. The study that observed a genotype effect on the bean root microbiota included wild, domesticated and landrace common beans of Mesoamerican origin that were grown in a greenhouse using Colombian field soil [6]. The authors attributed those bean genotype differences to differences in root architecture, which often has a different phenotype in greenhouse pots than in the field [8]. We summarize that, in this study and in agreement with other field studies, plant genotype has a minor to no measurable impact on rhizosphere microbiome.

Supporting Materials and Methods

Soil chemical analysis

Soil analysis was done at the Michigan State Soil and Plant Nutrient Laboratory by agricultural soil testing standard protocols and included following parameters: pH, phosphorous (P ppm), potassium (K ppm), calcium (Ca ppm), magnesium (Mg ppm), organic matter content (OM%), nitrate (NO_3^- ppm), ammonium (NH_4^+ ppm) and total nitrogen (%). pH was determined in water in a 1:2 ratio (soil:water) and measured with an AS3000 pH Analyzer. The Olsen phosphorus test was used to determine the phosphorus in the soil and measured using a Brinkmann PC950 Probe Colorimeter. Extractable K and Ca were determined by flame emission and Mg colorimetrically using the Seal AA3. The total organic matter was measured using the loss-on-ignition method.

Total N quantification was done by the Kjeldahl block digestion method [9]. Soil nitrate analysis was done using cadmium reduction and measured by Lachat QuikChem 8500 Flow Injection Analyzer. For ammonium, soils were first resuspended in KCl solution and the salicylate method was used to quantify the concentrations using the Lachat QuikChem 8500 Flow Injection Analyzer. This analysis was done only for the soil samples obtained from the biogeography experiment conducted in 2017, and measurements were done for each sample.

DNA isolation and sequencing

For the biogeography samples collected in 2017, DNA was isolated using DNeasy PowerSoil kit (QIAGEN, US) by following the manufacturer's recommendations. For the plant development samples collected in 2018, DNA was isolated using Griffiths protocol [10] except for the SVERC rhizosphere for which DNeasy PowerSoil kit (QIAGEN, US) was used because the Griffith protocol had low nucleic acid yield. Thus, we also assessed the effect of DNA isolation protocol on amplicon sequencing outcomes by comparing eight MRF rhizosphere samples for which DNA was isolated using both methods. Analyses suggest that the relative abundance of only 12 out of 48 core taxa was significantly influenced by the isolation method (**Fig. S1**) thus we believe that the comparison of relative abundance of core taxa, between the root compartments in SVERC is possible. Notably, isolation methods influenced both alpha (richness but not Shannon and Pielou) and beta diversity (**Fig. S2**).

Quality and quantity of isolated DNA was assessed with Qubit 2.0 fluorometer using HS dsDNA Assay kit (ThermoFisher, US). Presence of 16S rRNA genes was confirmed by PCR using the V4 16S rRNA sequencing primer set 515f and 806r [11] and then visualizing the PCR products by agarose gel electrophoresis. In 2017 for the spatial study, rhizosphere samples were pooled per location by field plot. In 2018 for the temporal study, rhizoplane and rhizosphere

samples were assessed for each individual plant harvested. 16S rRNA gene amplicon samples were prepared by the Michigan State Genomics Core Research Support Facility. Their standard protocol included 16S rRNA gene amplicon PCR amplification and library preparation. The ITS samples were amplified in our lab using the primer pair ITS1f and ITS2 [12] with index adapters as recommended Genomics Core (https://rtsf.natsci.msu.edu/genomics/sample-requirements/illumina-sequencing-sample-requirements/, June 2019), The Genomics Core used the Illumina TruSeq Nano DNA library preparation kit for both 16S and ITS libraries. Paired-end, 250-bp reads were generated on an Illumina MiSeq platform using v2 Standard 500 cycle kit, and the Genomics Core provided standard Illumina quality control, adaptor, barcode trimming, and sample demultiplexing with Illumina Bcl2fastq v2.19.1. In all sequencing efforts we included blank sample (negative control) which were processed the same way to identify OTUs resulting from contamination either through DNA isolation or PCR.

Statistical analysis

Alpha and beta diversity analyses were performed to datasets subsampled to the minimum observed quality filtered reads per sample (2017 dataset: 31,255 for 16S rRNA and 22,716 for ITS, 2018 dataset: 15,000 for 16S rRNA). Due to low read counts one sample (SVERC1) was removed from the 16S rRNA 2017 dataset. We report richness as total number of OTUs clustered at 97% sequence identity. Differences in alpha diversity among groups (plant genotype, root compartment, sampling time, growing location, fertilization, pH) were assessed using analysis of variance (ANOVA), with a Tukey *post hoc* test for multiple comparisons. We used the *protest* function in the vegan package in R [13] (version 2.5-6) to test for synchrony between bacterial/archaeal and fungal communities.

To quantify the variation in beta diversity (community structure), we first calculated pairwise Bray-Curtis dissimilarities. Permutational multivariate analysis (PERMANOVA) using 1,000 random permutations was used to test hypothesis of beta diversity using *adonis* function in the vegan package in R [13].

We used DESeq2 [5] to determine bacterial and archaeal taxa with differential abundance across plant genotypes from the biogeography study. Thresholds for calling taxa as differentially abundant between selected groups was set at p-value of 0.05.

We prioritized core taxa over space and time using abundance-occupancy distributions fitted to the Sloan neutral model, as recently described [14]. Species abundance-occupancy distributions are often applied to explore large-scale patterns in species distributions, especially in macroecology [15]. For that, first taxon's mean relative abundance is calculated across the dataset and log transformed and secondly, its frequency of detection across dataset is calculated (termed as occupancy, with 1 represented in all samples). In this study we considered as core all taxa that were found in every sample across growing locations. To assess the importance of neutral process in the assembly of common bean rhizosphere communities we applied the Sloan neutral model [16]. This model predicts the relationship between the frequency with which taxa occur in a set of local communities (occupancy) and their mean abundance across a broader metacommunity. The prediction is that rare taxa will be lost from individual hosts due to ecological drift, but abundant taxa will be more widespread in a metacommunity due to higher chance of dispersal and thus be randomly sampled by individual hosts. The occupancy of OTUs and their mean relative abundances across the metacommunity were fitted to the model, using the R code described by Burns et al. [17]. We used the following parameters of neutral model:

95% confidence intervals, the goodness of fit of the neutral model (R^2), and the estimated migration rate (m).

In addition to the Sloan neutral model, we also used the iCAMP to infer quantitatively the importance of assembly mechanisms [18]. This framework achieves this by phylogenetic-bin-based null model analysis. Since this analysis depends on phylogenetic information, we generated a phylogenetic tree from 16S OTU representative sequences obtained in the US biogeography study (2017). As, reference based and de-novo OTUs differed in length we first trimmed them to the same region with cutadapt [19] using EMP V4 primer sequences. Sequence alignment, gap removal, building and rooting the phylogenetic tree was all performed in QIIME2 environment [20]. Datasets were filtered in phyloseq [21] to resemble data used for the neutral modelling. We utilized the online version of the iCAMP tool (http://ieg3.rccc.ou.edu:8080/) using 8 threads and 1000-time randomization (max allowed). The outputs were processed in R.

Co-occurrence network analysis

Global network properties were calculated using the Molecular Ecological Network Analysis Pipeline (MENAP) [22]. The majority was set to 0.5, missing data were kept blank, read counts were converted by the logarithm, and the Pearson correlation coefficient was used as the similarity measure. The results were then filtered by keeping pairwise correlations with absolute LA values greater or equal to 0.88. To determine the modularity of the network we created 100 random networks within MENAP using the same number of nodes and edges as the complete potentially active network. The general properties of the inferred networks were analyzed using NetworkAnalyzer in Cytoscape [23, 24].

The connectivity of each node in the network was calculated using within-module connectivity (Zi) and among-module connectivity (Pi) scores which defined the topological role of each node (taxon) [22, 25]. We classified our nodes as per the four categories describing node topology: *network hubs* (highly connected nodes within the entire network, Zi > 2.5 and Pi > 0.62), *module hubs* (highly connected nodes within modules, Zi > 2.5), *connectors* (nodes that connect modules, Pi > 0.62), and *peripheral* nodes (nodes connected in modules with few outside connections, Zi < 2.5 and Pi < 0.62) [25].

Cytoscape v.3.5.1 [23] was used for visualization of significant co-occurrences and editing the appearance of nodes size, shape and color based on the number of connections (degrees), taxonomic affiliation and module, respectively.

Comparative analyses with published datasets

To determine whether members of the US bacterial and archaeal core were associated more generally with bean plants grown, we compared our data to a published study of bean rhizosphere conducted in Colombia that investigated the influence of common plant genotype and root architecture on the rhizosphere microbiome composition. This published study included eight Mesoamerican genotypes planted in an agricultural soil (Colombia forest), and grew the plants in the greenhouse [6] (NCBI BioProject ID PRJEB19467), We downloaded and processed raw reads as described above, and, despite differences in our processing pipeline and the originally published pipeline, we generated very similar numbers of OTUs (12,209 compared to 12,293 [6]). Because the studies used different sequencing primers, we identified identical OTUs by matching the IDs provided by the SILVA database v128 [26] for all reference clustered OTUs. For the *de-novo* clustered OTUs, we used BLAST [27] to identify 100% matches between

the two datasets, across the overlapping V4 region of reads from both studies. As before, we calculated occupancy of every taxon in the rhizosphere datasets and identified those with occupancy of 1. Taxa with the same OTU IDs or 100% BLAST match were designated as the cross-continental core rhizosphere microbiota of the common bean.

A similar approach was used to compare the data published in Pérez-Jaramillo et al. [28] (BioProject ID PRJEB26084). We combined this dataset with the one from above (PRJEB19467 [28]) and re-run the same UPARSE pipeline (version 11) [29] and classify the OTUs with SILVA database v128 [26]. We attempted to re-analyzed these data as closely as to what was published by the authors, however, due to incomplete methodology description and metadata, we were not able to do so. Thus, we applied these published data to search for the core taxa occupancy in agricultural and forest soils.

Supporting references

1. Walters WA, Jin Z, Youngblut N, Wallace JG, Sutter J, Zhang W, et al. Large-scale replicated field study of maize rhizosphere identifies heritable microbes. *Proc Natl Acad Sci U S A* 2018; **115**: 7368–7373.
2. Wagner MR, Lundberg DS, del Rio TG, Tringe SG, Dangl JL, Mitchell-Olds T. Host genotype and age shape the leaf and root microbiomes of a wild perennial plant. *Nat Commun* 2016; **7**: 12151.
3. Xu J, Zhang Y, Zhang P, Trivedi P, Riera N, Wang Y, et al. The structure and function of the global citrus rhizosphere microbiome. *Nat Commun* 2018; **9**: 4894.
4. Edwards J, Johnson C, Santos-Medellín C, Lurie E, Podishetty NK, Bhatnagar S, et al. Structure, variation, and assembly of the root-associated microbiomes of rice. *Proc Natl*

Acad Sci U S A 2015; **112**: E911-20.

5. Love MI, Huber W, Anders S. Moderated estimation of fold change and dispersion for RNA-seq data with DESeq2. *Genome Biol* 2014; **15**: 550.
6. Perez-Jaramillo JE, Carrion VJ, Bosse M, Ferrao LF V, de Hollander M, Garcia AAF, et al. Linking rhizosphere microbiome composition of wild and domesticated Phaseolus vulgaris to genotypic and root phenotypic traits. *ISME J* 2017; **11**: 2244–2257.
7. Edwards JA, Santos-Medellín CM, Liechty ZS, Nguyen B, Lurie E, Eason S, et al. Compositional shifts in root-associated bacterial and archaeal microbiota track the plant life cycle in field-grown rice. *PLOS Biol* 2018; **16**: e2003862.
8. Rich SM, Watt M. Soil conditions and cereal root system architecture: review and considerations for linking Darwin and Weaver. *J Exp Bot* 2013; **64**: 1193–1208.
9. Bradstreet R. The Kjeldahl Method for Organic Nitrogen. *The Kjeldahl Method for Organic Nitrogen* . 1965. Academic Press, New Yourk.
10. Griffiths RI, Whiteley AS, O'donnell AG, Bailey MJ. Rapid Method for Coextraction of DNA and RNA from Natural Environments for Analysis of Ribosomal DNA-and rRNA-Based Microbial Community Composition. *APPLIED AND ENVIRONMENTAL MICROBIOLOGY* . 2000.
11. Caporaso JG, Lauber CL, Walters WA, Berg-Lyons D, Lozupone CA, Turnbaugh PJ, et al. Global patterns of 16S rRNA diversity at a depth of millions of sequences per sample. *Proc Natl Acad Sci U S A* 2011; **108**: 4516–22.
12. White T, Bruns T, Lee S, Taylor FJRM, White T, Lee S-H, et al. Amplification and direct sequencing of fungal ribosomal RNA genes for phylogenetics. In: Innis M, Gelfand D, Sninsky J, White T (eds). *PCR - Protocols and Applications - A Laboratory Manual*, 1st

ed. 1990. Academic Press, pp 315–322.

13. Oksanen J, Blanchet FG, Friendly M, Kindt R, Legendre P, McGlinn D, et al. vegan: Community Ecology Package. 2017.

14. Shade A, Stopnisek N. Abundance-occupancy distributions to prioritize plant core microbiome membership. *Curr Opin Microbiol* 2019; **49**: 50–58.

15. Shade A, Dunn RR, Blowes SA, Keil P, Bohannan BJM, Herrmann M, et al. Macroecology to Unite All Life, Large and Small. *Trends Ecol Evol* 2018; **33**: 731–744.

16. Sloan WT, Lunn M, Woodcock S, Head IM, Nee S, Curtis TP. Quantifying the roles of immigration and chance in shaping prokaryote community structure. *Environ Microbiol* 2006; **8**: 732–740.

17. Burns AR, Stephens WZ, Stagaman K, Wong S, Rawls JF, Guillemin K, et al. Contribution of neutral processes to the assembly of gut microbial communities in the zebrafish over host development. *ISME J* 2016; **10**: 655–664.

18. Ning D, Yuan M, Wu L, Zhang Y, Guo X, Zhou X, et al. A quantitative framework reveals ecological drivers of grassland microbial community assembly in response to warming. *Nat Commun* 2020; **11**: 1–12.

19. Martin M. Cutadapt removes adapter sequences from high-throughput sequencing reads. *EMBnet.journal* 2011; **17**: 10.

20. Bolyen E, Rideout JR, Dillon MR, Bokulich NA, Abnet CC, Al-Ghalith GA, et al. Reproducible, interactive, scalable and extensible microbiome data science using QIIME 2. *Nat Biotechnol* . 2019. Nature Publishing Group. , **37**: 852–857

21. Callahan BJ, Sankaran K, Fukuyama JA, Mcmurdie PJ, Holmes SP, Lahti L, et al. Bioconductor workflow for microbiome data analysis: from raw reads to community

analyses. *F1000Research* 2016; **898618986**: 1492–21492.

22. Deng Y, Jiang Y-H, Yang Y, He Z, Luo F, Zhou J. Molecular ecological network analyses. *BMC Bioinformatics* 2012; **13**: 113.

23. Shannon P, Markiel A, Ozier O, Baliga NS, Wang JT, Ramage D, et al. Cytoscape: A Software Environment for Integrated Models of Biomolecular Interaction Networks. *Genome Res* 2003; **13**: 2498–2504.

24. Doncheva NT, Assenov Y, Domingues FS, Albrecht M. Topological analysis and interactive visualization of biological networks and protein structures. *Nat Protoc* 2012; **7**: 670–685.

25. Guimerà R, Nunes Amaral LA. Functional cartography of complex metabolic networks. *Nature* 2005; **433**: 895–900.

26. Quast C, Pruesse E, Yilmaz P, Gerken J, Schweer T, Yarza P, et al. The SILVA ribosomal RNA gene database project: improved data processing and web-based tools. *Nucleic Acids Res* 2013; **41**: D590-6.

27. Madden T. The BLAST Sequence Analysis Tool. 2013.

28. Pérez-Jaramillo JE, de Hollander M, Ramírez CA, Mendes R, Raaijmakers JM, Carrión VJ. Deciphering rhizosphere microbiome assembly of wild and modern common bean (Phaseolus vulgaris) in native and agricultural soils from Colombia. *Microbiome* 2019; **7**: 114.

29. Edgar RC. UPARSE: highly accurate OTU sequences from microbial amplicon reads. *Nat Methods* 2013; **10**: 996–998.

Supporting Figure legends

Fig. S1: Comparison of relative abundance of the 48 core OTUs between the DNA isolation methods used in the development study (G=Griffith, P=PowerSoil). Statistical difference, determined by Wilcoxon test, is represented as star symbol (*<0.05, **<0.01, ***<0.001). The pints are color coded by their original sample.

Fig. S2: The effect of isolation method on alpha and beta diversity. For alpha diversity richness, Shannon and Pielou indices are presented (A). For the principal coordinates analysis, Bray-Curtis distance matrix was used. Symbols are colored by samples. Wilcoxon test was used to determine statistical differences between isolation methods for the alpha diversity metrices (*<0.05). PERMANOVA was used to determine the effect of isolation method on community structure.

Fig. S3: Sequencing depth and rarefaction curves for 16S rRNA (A,B) and ITS (C, D) dataset from samples collected in 2017. The red dashed line represents the rarefaction threshold. There was sample to sample variation in read depth within the dataset because we used multiple sequencing efforts, and these efforts had different numbers of samples included in the multiplexing, resulting in different numbers of returned reads per sample; specifically the rhizosphere 16S rRNA samples were multiplexed and sequenced separately from the ITS and root-associated 16S rRNA samples. Despite these differences, the rarefactions show that the sequencing effort was satisfactory for all samples included.

Fig. S4: Alpha diversity indices for the 16S RNA (A, B) and ITS (C, D) datasets. Represented are rich-ness, Shannon and Pielou indices measured by growing location (A, C) and bean genotype (B, D). For statistical comparison of the pairs we used ANOVA (A, C) or Wilcoxon test (B, D).

Fig. S5: Growing location drives bacterial/archaeal (A) and fungal (B) microbiome structure of the common bean rhizosphere. The principal coordinate analysis (PCoA) is based on Bray-Curtis distances. Growing location is indicated by color and plant genotype is indicated by shape shapes (diamond=CELRK, circle=Eclipse, square=bulk soil). The strength of statistically significant (p-value < 0.01) explanatory variables are shown as the length of fitted vectors.

Fig S6: Community composition (A, C) and number of shared taxa between sites represent as Venn diagrams (B, D). The 16S rRNA dataset is represented in the top panels (A, B) and ITS in the lower panels (C, D). The bar charts are colored based on the phylum association (phyla represented by relative abundance < .05 are grouped and labelled as other). For Venn diagrams, samples were grouped by the growing location and bulk soil samples were removed.

Fig. S7: Quantification of assembly processes as predicted by the iCAMP tool [38]. The mean of relative importance of deterministic (homogeneous (HoS) and heterogeneous selection (HeS)) and stochastic (homogenizing dispersal (HD), dispersal limitation (DL), and drift) processes is 40.4% and 59.6% across sites, respectively (A). Overall, 48 core taxa were binned into 35 phylogenetic bins, from which 23 bins were represented by a core taxon as the top taxon (highest relative abundance from the iCAMP output). A relatively higher importance of stochastic

mechanisms over deterministic was predicted for 11 bins containing 25 core taxa (B). The relative importance of stochastic assembly is consistent (in all locations) for 7 core taxa, but for the rest of the core, it is less consistent (C). The taxonomic affiliation is highlighted only for core taxa predicted to be deterministically assembled, and of these, all were attributed to homogenizing selection.

Fig. S8: Analysis of ZOTUs represented by each identified core OTU. The biogeography dataset was used to generate ZOTUs using UNOISE pipeline. 48 core OTUs were represented by as few as 2 ZOTUs and by up to 35 ZOTUs. For every OTU we found at least two ZOTUs with occupancy = 1 and all of them, except of 2 ZOTUs, had also the highest relative abundance among them. Points are color coded by their presence, red representing those with occupancy < 1 and blue for ZOTUs with occupancy of 1. The OTUs on the x-axis are ordered alphabetically and colored as classified at the phylum level. For detailed taxonomic classification of the core taxa please see Table S4.

Fig. S9: Occupancy of core OTUs in the development study. Occupancy is represented by color and size. The x-axis labels correspond to the following bean developmental stages: 1 – V2, 2 – V5, 3 – flowering, 4 – senescence and 6 – dry.

Supporting Table legends

Table S1: PERMANOVA results for the 16S rRNA and ITS data. Highly correlated or/and statistically significant values are highlighted in bold.

Table S2: 20 differentially abundant OTUs between the two plant genotypes as identified by using DESeq2 (Love et al. 2014).

Table S3: Sloan neutral model summary.

Table S4: List of 48 core taxa as defined from the meta-analysis and their taxonomic classification as by the SILVA database v128 (Quast et al. 2013).

Table S5: Occupancy of core OTUs in agricultural, natural (forest) soils or when combined. Results are based on the re-analysis of the data from the Pérez-Jaramillo et al. 2019. OTUs with occupancy of 1 in both soils are highlighted in orange. Additional to the number we used green gradient shading to represents the occupancy of each OTU.

Table S6: Summary of network properties of actual and randomly generated networks.

Figure S1

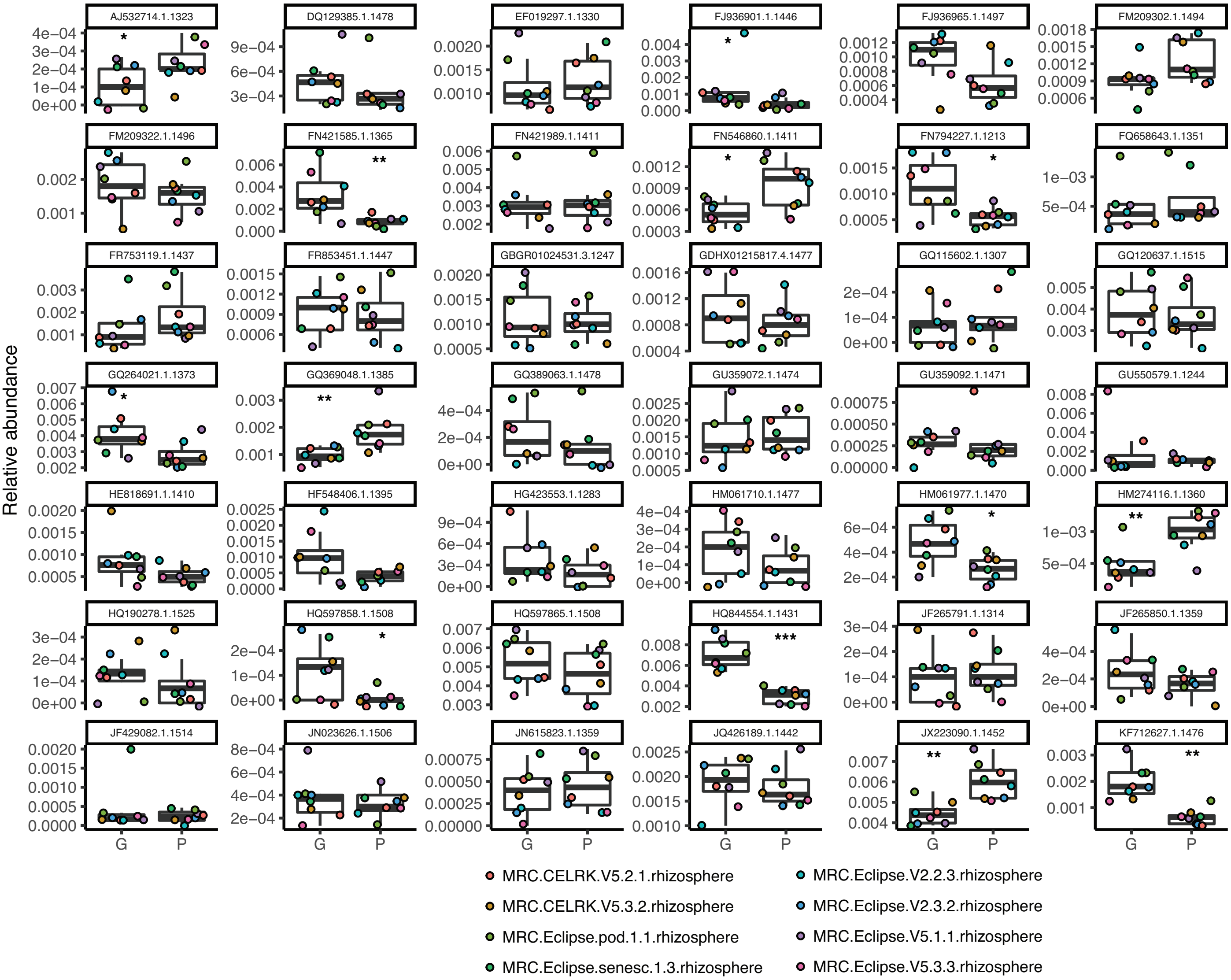

Figure S2

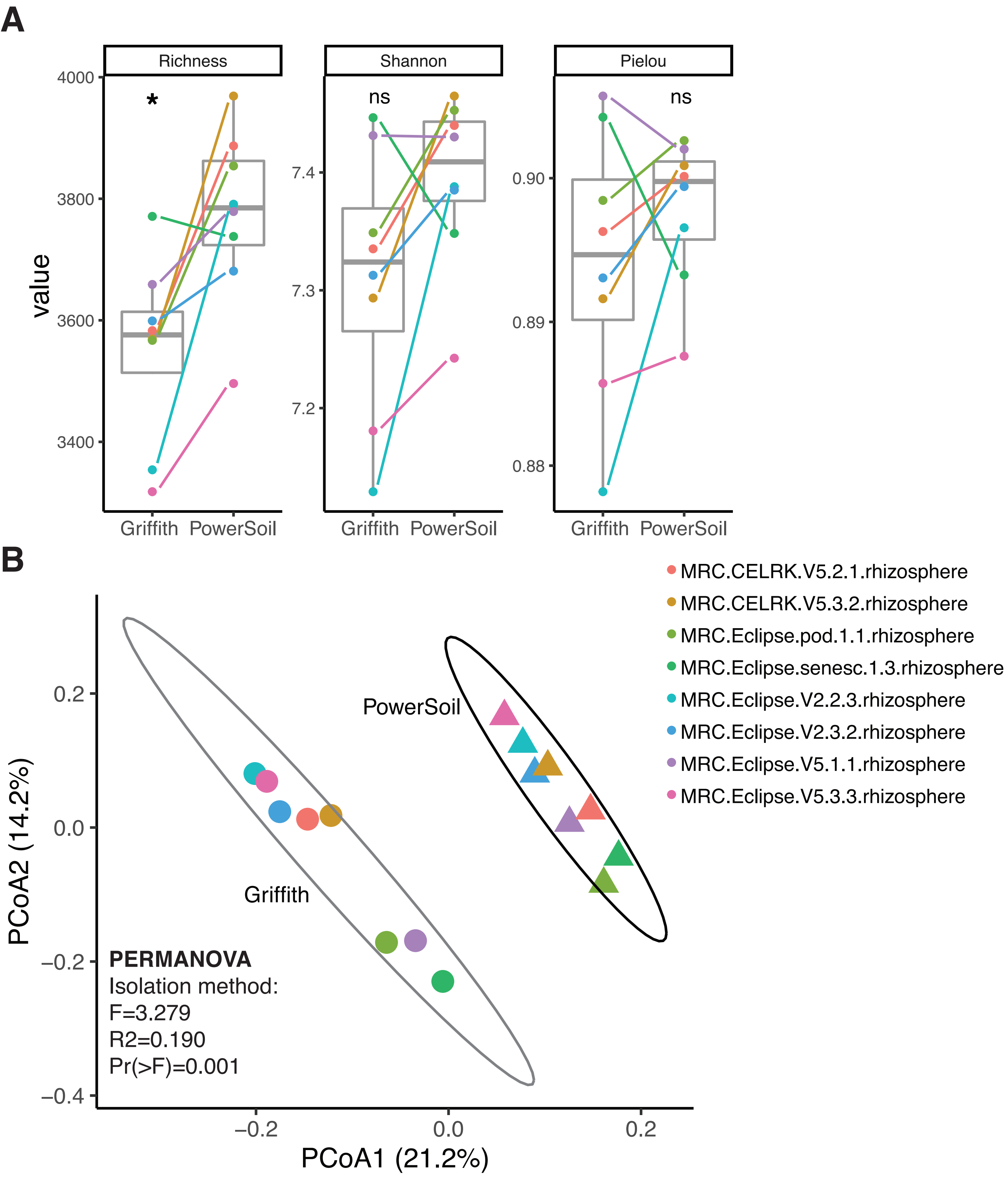
A
Richness
Shannon
Pielou
value
*
ns
ns
4000
3800
3600
3400
7.4
7.3
7.2
0.90
0.89
0.88
Griffith
PowerSoil
B
PowerSoil
Griffith
PERMANOVA
Isolation method:
F=3.279
R2=0.190
Pr(>F)=0.001
PCoA2 (14.2%)
PCoA1 (21.2%)
0.2
0.0
−0.2
−0.4
MRC.CELRK.V5.2.1.rhizosphere
MRC.CELRK.V5.3.2.rhizosphere
MRC.Eclipse.pod.1.1.rhizosphere
MRC.Eclipse.senesc.1.3.rhizosphere
MRC.Eclipse.V2.2.3.rhizosphere
MRC.Eclipse.V2.3.2.rhizosphere
MRC.Eclipse.V5.1.1.rhizosphere
MRC.Eclipse.V5.3.3.rhizosphere

Figure S3

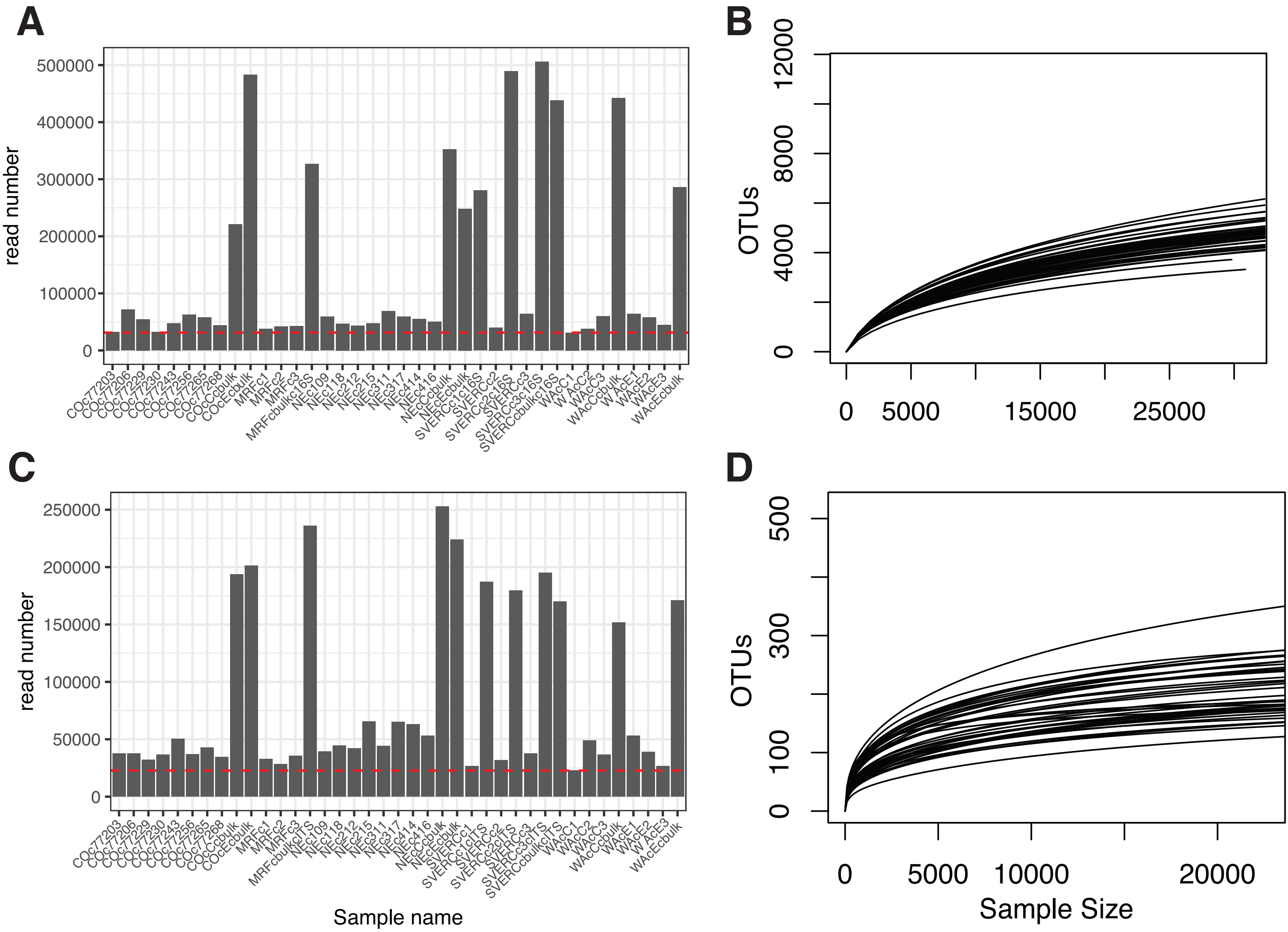

Figure S4

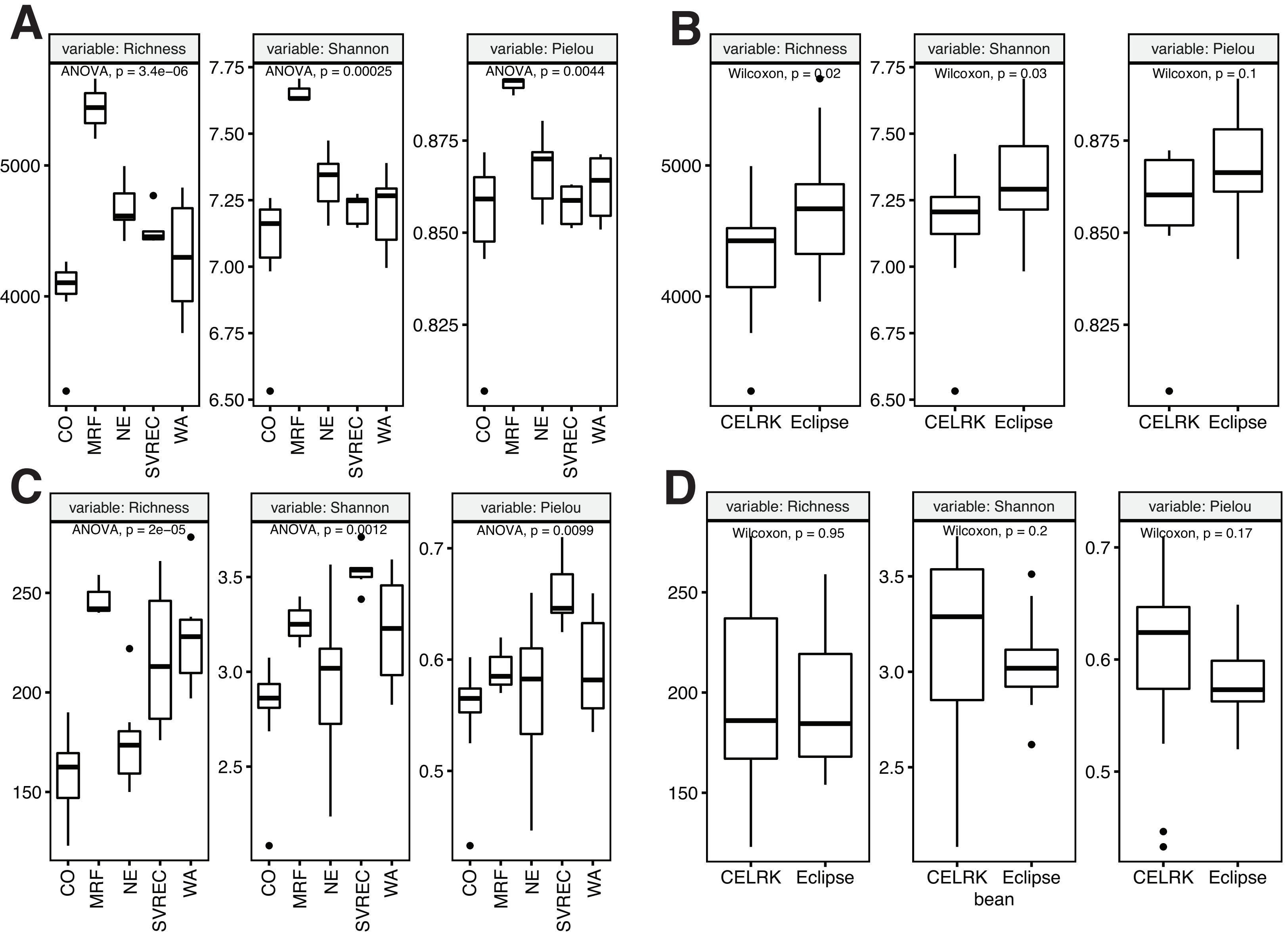
A
B
C
D
variable: Richness
variable: Shannon
variable: Pielou
ANOVA, p = 3.4e−06
ANOVA, p = 0.00025
ANOVA, p = 0.0044
Wilcoxon, p = 0.02
Wilcoxon, p = 0.03
Wilcoxon, p = 0.1
ANOVA, p = 2e−05
ANOVA, p = 0.0012
ANOVA, p = 0.0099
Wilcoxon, p = 0.95
Wilcoxon, p = 0.2
Wilcoxon, p = 0.17
CO
MRF
NE
SVREC
WA
CELRK
Eclipse
bean

Figure S5

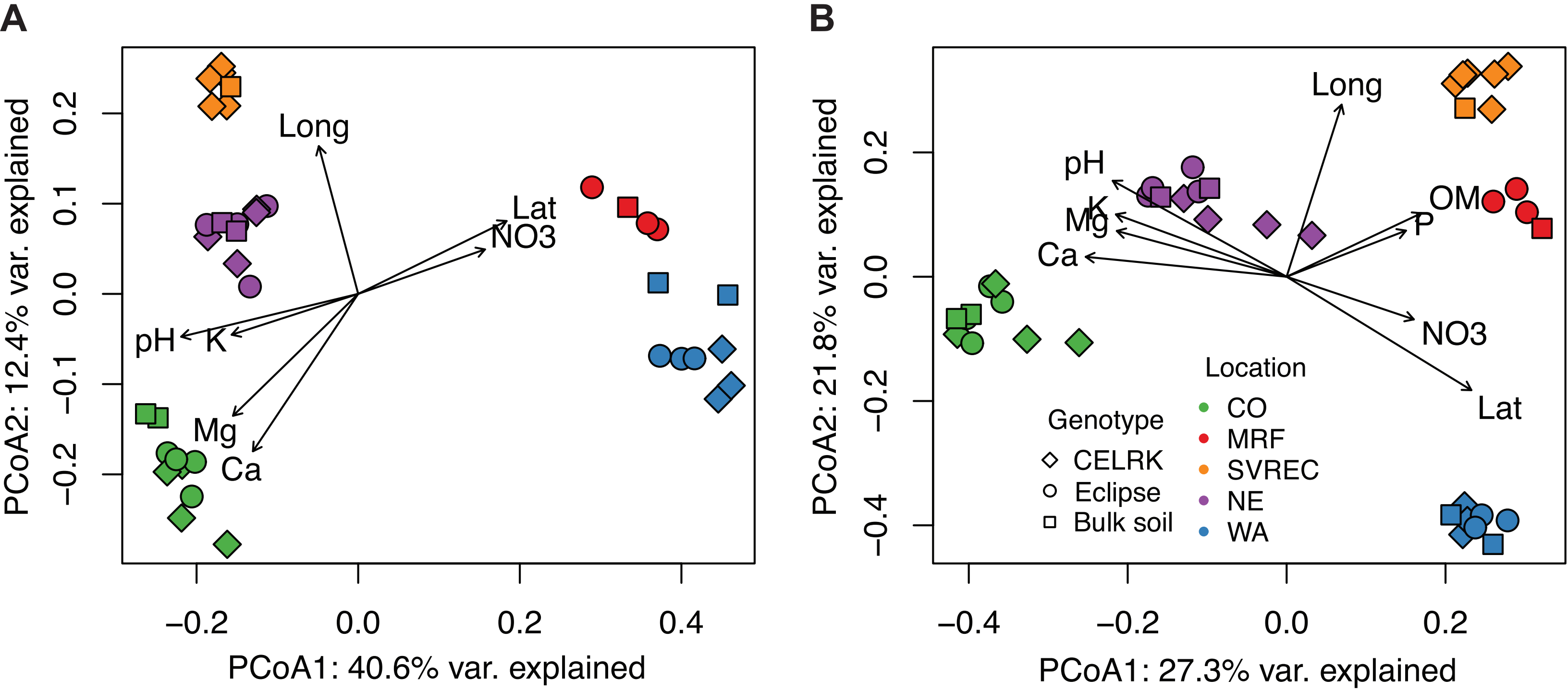
A
Long
Lat
NO3
pH
K
Mg
Ca
PCoA1: 40.6% var. explained
PCoA2: 12.4% var. explained
B
Long
pH
K
Mg
Ca
OM
P
NO3
Lat
Genotype
CELRK
Eclipse
Bulk soil
Location
CO
MRF
SVREC
NE
WA
PCoA1: 27.3% var. explained
PCoA2: 21.8% var. explained

Figure S6

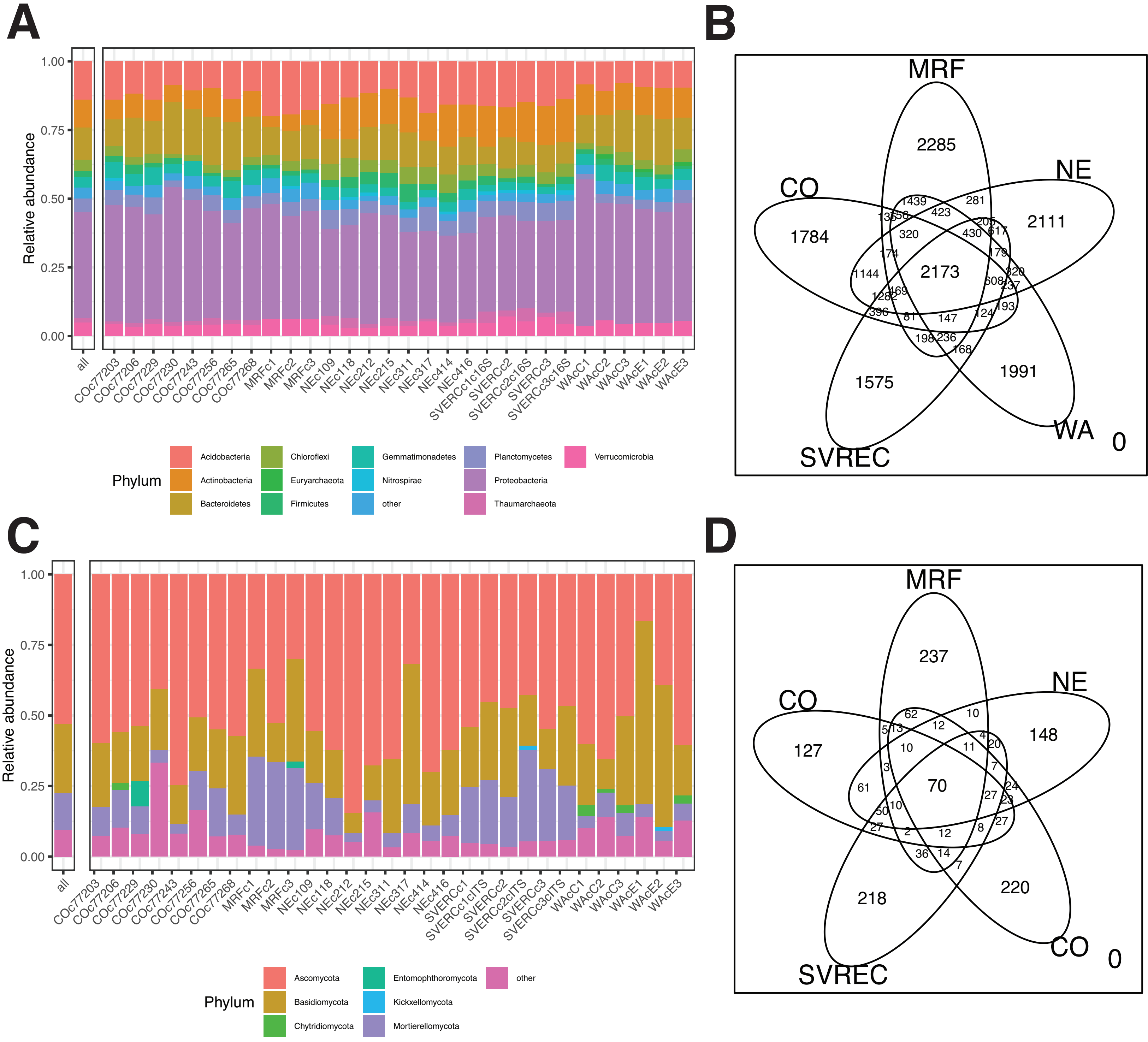

A
B
C
D
Relative abundance
1.00
0.75
0.50
0.25
0.00
all
COc77203
COc77206
COc77229
COc77230
COc77243
COc77256
COc77265
COc77268
MRFc1
MRFc2
MRFc3
NEc109
NEc118
NEc212
NEc215
NEc311
NEc317
NEc414
NEc416
SVERCc1c16S
SVERCc2
SVERCc2c16S
SVERCc3
SVERCc3c16S
WAcC1
WAcC2
WAcC3
WAcE1
WAcE2
WAcE3
Phylum
Acidobacteria
Actinobacteria
Bacteroidetes
Chloroflexi
Euryarchaeota
Firmicutes
Gemmatimonadetes
Nitrospirae
other
Planctomycetes
Proteobacteria
Thaumarchaeota
Verrucomicrobia
MRF
NE
CO
WA
SVREC
2285
1784
2111
2173
1575
1991
0
SVERCc1
SVERCc1cITS
SVERCc2cITS
SVERCc3cITS
Ascomycota
Basidiomycota
Chytridiomycota
Entomophthoromycota
Kickxellomycota
Mortierellomycota
237
127
148
70
218
220

Figure S7

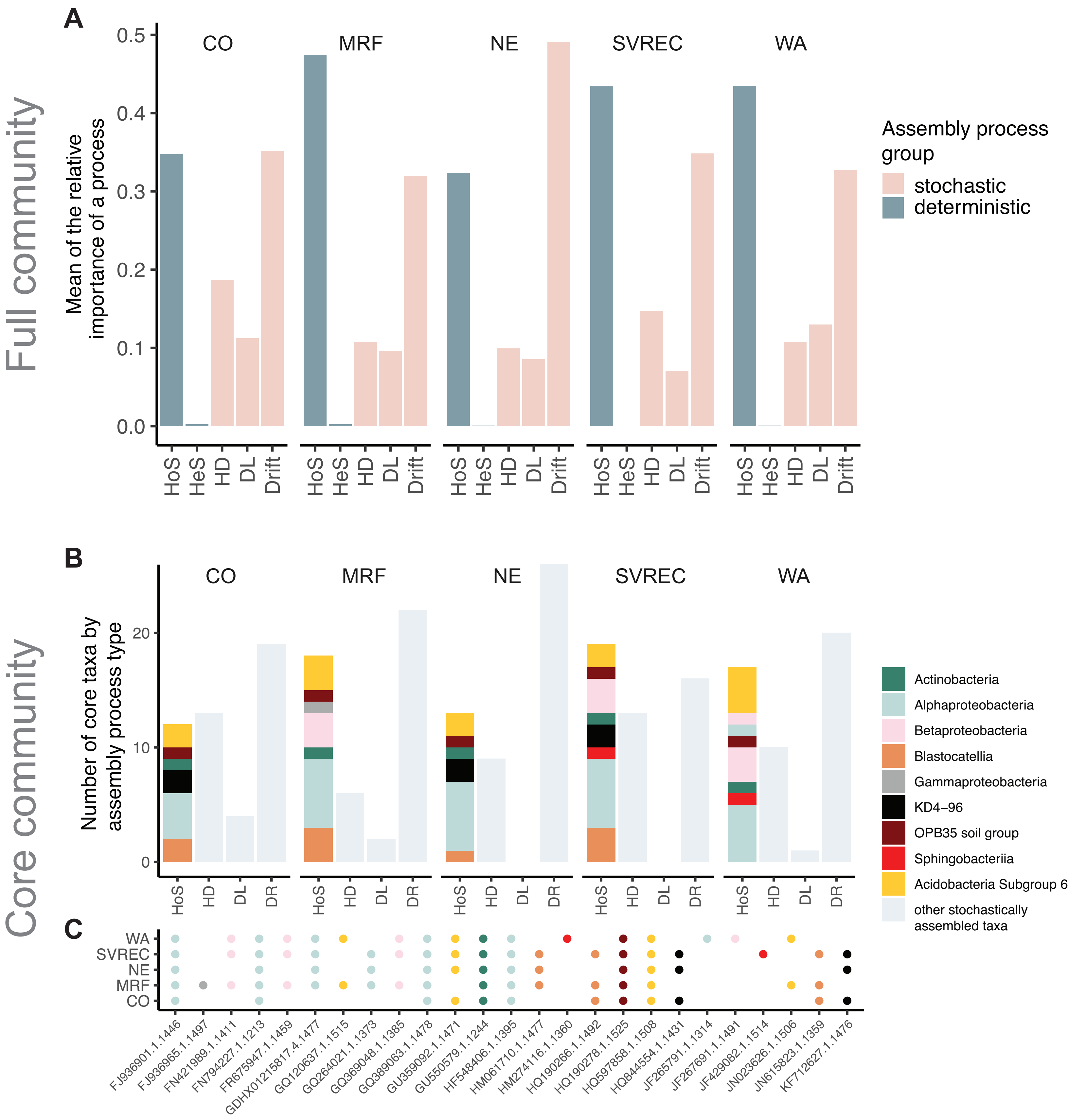
A
B
C
Full community
Core community
Mean of the relative importance of a process
Number of core taxa by assembly process type
CO
MRF
NE
SVREC
WA
HoS
HeS
HD
DL
Drift
DR
Assembly process group
stochastic
deterministic
Actinobacteria
Alphaproteobacteria
Betaproteobacteria
Blastocatellia
Gammaproteobacteria
KD4–96
OPB35 soil group
Sphingobacteriia
Acidobacteria Subgroup 6
other stochastically assembled taxa
FJ936901.1.1446
FJ936965.1.1497
FN421989.1.1411
FN794227.1.1213
FR675947.1.1459
GDHX01215817.4.1477
GQ120637.1.1515
GQ264021.1.1373
GQ369048.1.1385
GQ389063.1.1478
GU359092.1.1471
GU550579.1.1244
HF548406.1.1395
HM061710.1.1477
HM274116.1.1360
HQ190266.1.1492
HQ190278.1.1525
HQ597858.1.1508
HQ844554.1.1431
JF265791.1.1314
JF267691.1.1491
JF429082.1.1514
JN023626.1.1506
JN615823.1.1359
KF712627.1.1476

Figure S8

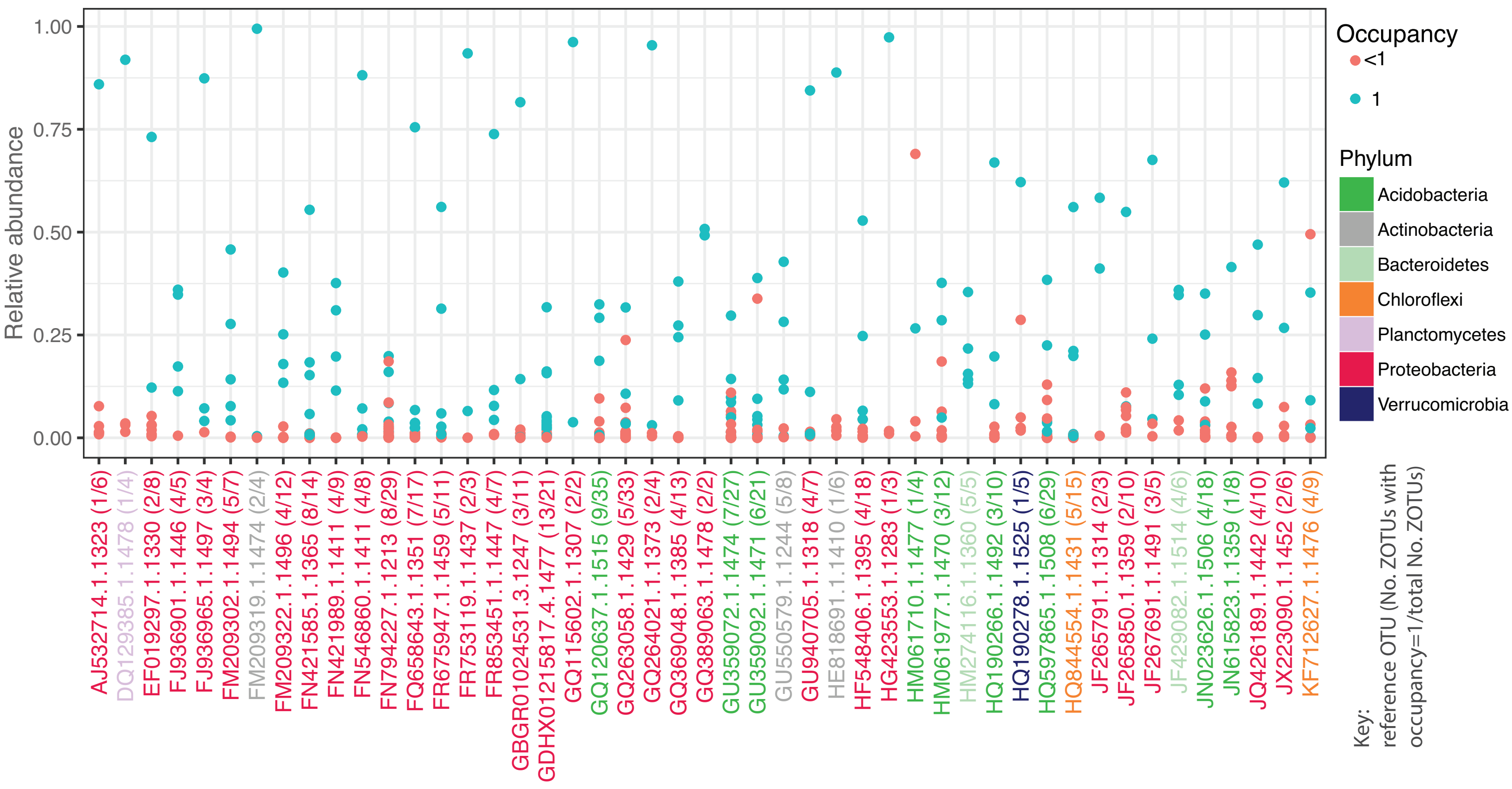

Relative abundance
1.00
0.75
0.50
0.25
0.00
Occupancy
<1
1
Phylum
Acidobacteria
Actinobacteria
Bacteroidetes
Chloroflexi
Planctomycetes
Proteobacteria
Verrucomicrobia
AJ532714.1.1323 (1/6)
DQ129385.1.1478 (1/4)
EF019297.1.1330 (2/8)
FJ936901.1.1446 (4/5)
FJ936965.1.1497 (3/4)
FM209302.1.1494 (5/7)
FM209319.1.1474 (2/4)
FM209322.1.1496 (4/12)
FN421585.1.1365 (8/14)
FN421989.1.1411 (4/9)
FN546860.1.1411 (4/8)
FN794227.1.1213 (8/29)
FQ658643.1.1351 (7/17)
FR675947.1.1459 (5/11)
FR753119.1.1437 (2/3)
FR853451.1.1447 (4/7)
GBGR01024531.3.1247 (3/11)
GDHX01215817.4.1477 (13/21)
GQ115602.1.1307 (2/2)
GQ120637.1.1515 (9/35)
GQ263058.1.1429 (5/33)
GQ264021.1.1373 (2/4)
GQ369048.1.1385 (4/13)
GQ389063.1.1478 (2/2)
GU359072.1.1474 (7/27)
GU359092.1.1471 (6/21)
GU550579.1.1244 (5/8)
GU940705.1.1318 (4/7)
HE818691.1.1410 (1/6)
HF548406.1.1395 (4/18)
HG423553.1.1283 (1/3)
HM061710.1.1477 (1/4)
HM061977.1.1470 (3/12)
HM274116.1.1360 (5/5)
HQ190266.1.1492 (3/10)
HQ190278.1.1525 (1/5)
HQ597865.1.1508 (6/29)
HQ844554.1.1431 (5/15)
JF265791.1.1314 (2/3)
JF265850.1.1359 (2/10)
JF267691.1.1491 (3/5)
JF429082.1.1514 (4/6)
JN023626.1.1506 (4/18)
JN615823.1.1359 (1/8)
JQ426189.1.1442 (4/10)
JX223090.1.1452 (2/6)
KF712627.1.1476 (4/9)
Key:
reference OTU (No. ZOTUs with
occupancy=1/total No. ZOTUs)

Figure S9

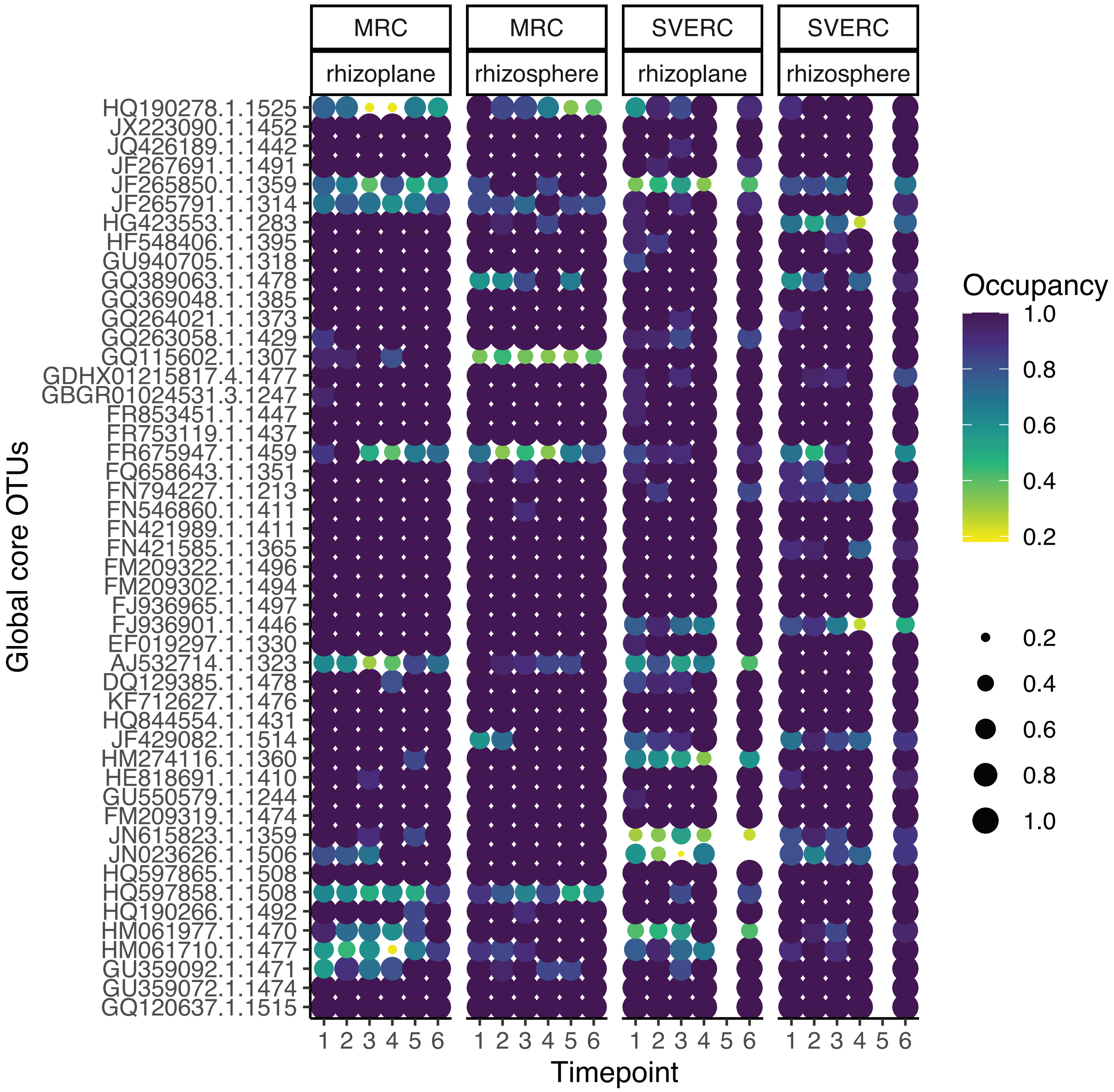
MRC
rhizoplane
MRC
rhizosphere
SVERC
rhizoplane
SVERC
rhizosphere
Global core OTUs
HQ190278.1.1525
JX223090.1.1452
JQ426189.1.1442
JF267691.1.1491
JF265850.1.1359
JF265791.1.1314
HG423553.1.1283
HF548406.1.1395
GU940705.1.1318
GQ389063.1.1478
GQ369048.1.1385
GQ264021.1.1373
GQ263058.1.1429
GQ115602.1.1307
GDHX01215817.4.1477
GBGR01024531.3.1247
FR853451.1.1447
FR753119.1.1437
FR675947.1.1459
FQ658643.1.1351
FN794227.1.1213
FN546860.1.1411
FN421989.1.1411
FN421585.1.1365
FM209322.1.1496
FM209302.1.1494
FJ936965.1.1497
FJ936901.1.1446
EF019297.1.1330
AJ532714.1.1323
DQ129385.1.1478
KF712627.1.1476
HQ844554.1.1431
JF429082.1.1514
HM274116.1.1360
HE818691.1.1410
GU550579.1.1244
FM209319.1.1474
JN615823.1.1359
JN023626.1.1506
HQ597865.1.1508
HQ597858.1.1508
HQ190266.1.1492
HM061977.1.1470
HM061710.1.1477
GU359092.1.1471
GU359072.1.1474
GQ120637.1.1515
1 2 3 4 5 6
Timepoint
Occupancy
1.0
0.8
0.6
0.4
0.2
0.2
0.4
0.6
0.8
1.0

Table S1: PERMANOVA results for the 16S rRNA and ITS data. Highly correlated or/and statistical significant values are highlighted in bold.

**Factor**	**PERMANOVA**			
	**16S rRNA**		**ITS**	
	R^2	Pr(>F)	R^2	Pr(>F)
pH	**0.3708**	**0.001**	**0.2045**	**0.001**
sampling location	**0.6643**	**0.001**	**0.6219**	**0.001**
genotype	0.0419	0.147	0.0337	0.213
genotype X sampling location	0.0300	0.114	0.0377	**0.038**
irrigation	0.1205	**0.001**	0.1615	**0.001**
fertilization	**0.3550**	**0.001**	**0.3495**	**0.001**
soil source (bulk/rhizosphere)	0.0189	0.652	0.0161	0.809
P	0.1445	**0.001**	0.1244	**0.001**
Nitrogen	0.1138	**0.001**	0.0953	**0.001**
OM	0.0922	**0.005**	0.1414	**0.001**
NO3	**0.2127**	**0.001**	0.1151	**0.002**
NH4	0.0488	0.082	0.0586	**0.026**

Table S2: 20 differentially abundant OTUs between the two plant genotypes as identified by using DESeq2 (Love et al. 2014).

	baseMean	log2FoldChange	lfcSE	stat	pvalue	padj	otu_id	Kingdom	Phylum	Class	Order	Family	Genus	Species	otu
1	38.0704592	-1.451779782	0.37954541	-3.825049	0.00013075	0.03919114	OTU00424	Bacteria	Verrucomicrobia	OPB35 soil group	uncultured bacterium	uncultured bacterium	uncultured bacterium	uncultured bacterium	HQ190278.1.1525
2	25.1812957	-1.916389303	0.45877689	-4.1771706	2.95E-05	0.02573169	OTU00617	Bacteria	Verrucomicrobia	OPB35 soil group					JF180093.1.1374
3	38.4262664	-3.904691854	0.98245124	-3.9744383	7.05E-05	0.02750427	OTU00961	Bacteria	Proteobacteria	Alphaproteobacteria	Rhodospirillales	Rhodospirillaceae	Skermanella	Ambiguous_taxa	JX489944.1.1458
4	48.3975041	-3.276868794	0.85230293	-3.8447231	0.00012069	0.03919114	OTU01296	Bacteria	Actinobacteria	Thermoleophilia	Gaiellales	uncultured	uncultured microorganism	uncultured microorganism	JN387436.1.1303
5	9.6855234	-4.093919005	1.09342083	-3.7441385	0.00018101	0.04569169	OTU02717	Bacteria	Actinobacteria	Thermoleophilia	Solirubrobacterales	Elev-16S-1332	uncultured bacterium	uncultured bacterium	EU132641.1.1305
6	19.5200132	-3.371795219	0.84362594	-3.9967894	6.42E-05	0.02750427	OTU03595	Bacteria	Actinobacteria	Thermoleophilia	Gaiellales	uncultured	uncultured bacterium	uncultured bacterium	JN002796.1.1337
7	11.8818931	5.023503714	1.2563172	3.99859503	6.37E-05	0.02750427	OTU03919	Bacteria	Acidobacteria	Blastocatellia	Blastocatellales	Blastocatellaceae (Subgroup 4)	uncultured	uncultured bacterium	HM444976.1.1326
8	29.251818	-4.09681156	0.93776525	-4.3686963	1.25E-05	0.01998181	OTU04022	Bacteria	Tectomicrobia	uncultured bacterium	uncultured bacterium	uncultured bacterium	uncultured bacterium	uncultured bacterium	EU133902.1.1441
9	76.1441579	-6.161840492	1.19762511	-5.1450495	2.67E-07	0.00128269	OTU04087	Archaea	Thaumarchaeota	Soil Crenarchaeotic Group(SCG)					KJ566508.1.952
10	4.5968289	3.498571348	0.88319485	3.96126784	7.46E-05	0.02750427	OTU04457	Bacteria	Bacteroidetes	Cytophagia	Cytophagales	Cytophagaceae	Emticicia	uncultured Bacteroidetes bacterium	GQ870456.1.1465
11	61.9297391	-6.091022664	1.3110812	-4.6458012	3.39E-06	0.00812344	OTU04555	Bacteria	Proteobacteria	Deltaproteobacteria	Desulfurellales	Desulfurellaceae	H16	uncultured bacterium	KR779707.1.1373
12	11.8743863	-5.065575262	1.36043844	-3.7234873	0.00019649	0.04711824	OTU07166	Bacteria	Actinobacteria	Thermoleophilia	Gaiellales	uncultured	uncultured bacterium	uncultured bacterium	HQ011679.1.1209
13	13.5093406	3.583028623	0.90355746	3.96546846	7.33E-05	0.02750427	OTU07414	Bacteria	Proteobacteria	Alphaproteobacteria	Rhizobiales	Xanthobacteraceae	Pseudolabrys	Ambiguous_taxa	HM447715.1.1442
14	16.8087098	4.862880224	1.16833369	4.16223571	3.15E-05	0.02573169	OTU08904	Bacteria	Acidobacteria	Blastocatellia	Blastocatellales	Blastocatellaceae (Subgroup 4)	Stenotrophobacter		GQ487927.1.1485
15	6.76293453	-5.57113906	1.46788249	-3.7953577	0.00014743	0.04154408	OTU11276	Bacteria	Actinobacteria	Acidimicrobiia	Acidimicrobiales	uncultured	uncultured bacterium	uncultured bacterium	KC554100.1.1512
16	17.7166328	-4.829752254	1.27722376	-3.7814457	0.00015592	0.04154408	OTU11479	Bacteria	Tectomicrobia	uncultured bacterium	uncultured bacterium	uncultured bacterium	uncultured bacterium	uncultured bacterium	AY326516.1.1483
17	4.95153605	-5.335406781	1.29719927	-4.1130202	3.91E-05	0.02675594	OTU11500	Bacteria	Acidobacteria	Subgroup 6	Ambiguous_taxa	Ambiguous_taxa	Ambiguous_taxa	Ambiguous_taxa	EU131958.1.1382
18	3.56734995	4.98215484	1.2351709	4.03357532	5.49E-05	0.02750427	OTU13551	Bacteria	Ignavibacteriae	Ignavibacteria	Ignavibacteriales	BSV26	uncultured bacterium	uncultured bacterium	JN038960.1.1495
19	9.31133972	6.136954425	1.47615816	4.15738273	3.22E-05	0.02573169	OTU13670	Bacteria	Verrucomicrobia	Spartobacteria	Chthoniobacterales	DA101 soil group	Ambiguous_taxa	Ambiguous_taxa	EU135481.1.1380
20	5.15897262	4.301994784	1.12406001	3.82719315	0.00012961	0.03919114	OTU13714	Bacteria	Bacteroidetes	Sphingobacteriia	Sphingobacteriales	Chitinophagaceae	Ferruginibacter	Ambiguous_taxa	JQ087141.1.1447

Table S3: Sloan neutral model summary.

Parameters	16S rRNA	ITS
m	0.3008	0.0030
m.ci	0.0072	0.0004
m.mle	0.3008	0.0030
maxLL	-15698.61	-531.06
binoLL	-12020.22	204.39
poisLL	-12020.51	204.38
Rsqr	0.7425	0.3069
Rsqr.bino	0.4631	-2.9593
Rsqr.pois	0.4631	-2.9592
RMSE	0.1181	0.1606
RMSE.bino	0.1705	0.3839
RMSE.pois	0.1705	0.3839
AIC	-31393.22	-1058.12
BIC	-31377.23	-1047.78
AIC.bino	-24036.45	412.78
BIC.bino	-24020.46	423.11
AIC.pois	-24037.02	412.76
BIC.pois	-24021.04	423.09
N	31255	22716
Samples	30	31
Richness	21881	1295
Detect	3.20E-05	4.40E-05
